# Biomimetic nanodrug blocks CD73 to inhibit adenosine and boosts antitumor immune response synergically with photothermal stimulation

**DOI:** 10.1186/s12951-024-02487-4

**Published:** 2024-04-30

**Authors:** Tan Li, Xingyu Zhang, Chengyu Shi, Qiao Liu, Yuetao Zhao

**Affiliations:** Department of Biochemistry and Molecular Biology, School of Life Sciences, Central South University, Changsha, 410012 China

**Keywords:** Biomimetic photothermal nanomaterials, Black phosphorus, Tumor immunotherapy, Adenosine, Photothermal therapy

## Abstract

Combination of tumor immunotherapy with photothermal therapy (PTT) is a feasible tactic to overcome the drawback of immunotherapy such as poor immune response. *Via* triggering the immunogenic cells death (ICD), PTT can stimulate the activity of immune cells, but meanwhile, the level of adenosine is elevated *via* the CD73-induced decomposition of ATP which is overexpressed accompanying with the PTT process, resulting in negative feedback to impair the immune stimulation. Herein, we developed a novel biomimetic photothermal nanodrug to specifically block CD73 for inhibition of adenosine production and more efficient priming of the suppressive immune microenvironments. The nanodrug, named as AptEM@CBA, is constructed by encapsulation of photothermal agent black phosphorus quantum dots (BPQDs) and selective CD73 inhibitor α, β-Methyleneadenosine 5′-diphosphate (AMPCP) in chitosan nanogels, which are further covered with aptamer AS1411 modified erythrocyte membrane (EM) for biomimetic camouflage. With AS1411 induced active targeting and EM induced long blood circulation time, the enrichment of the nanodrug tumor sites is promoted. The photothermal treatment promotes the maturation of dendritic cells. Meanwhile, the release of AMPCP suppress the adenosine generation *via* CD73 blockade, alleviating the impairment of adenosine to dendritic cells and suppressing regulatory T cells, synergically stimulate the activity of T cells. The combination of CD73 blockade with PTT, not only suppresses the growth of primary implanted tumors, but also boosts strong antitumor immunity to inhibit the growth of distal tumors, providing good potential for tumor photoimmunotherapy.

**Supplementary Information:**

The online version contains supplementary material available at 10.1186/s12951-024-02487-4.

## Introduction

The incidence and mortality of lung cancer is one of the leading cause threatening humans’ healthy. Many efforts have been made for the treatment of lung cancer, including the “hotspot” immunotherapy, which primarily relies on the immune checkpoint blockade (ICB) against programmed cell death protein 1 (PD-1) and programmed death-ligand 1 (PD-L1). Although the clinical results are encouraging, immune checkpoint blockade (ICB) still faces obstacles like low immunological response (40–50%) and low anticancer effectiveness, owing to the inadequate immune-stimulative signals and the immune-suppressive factors in the tumor microenvironment (TME). Stimulation of immune signals and modulation of suppressive factors offer promising strategy to promote immune response against tumors and enhance the efficiency of immunotherapy [1–4].

Tumor phototherapy including photothermal therapy (PTT) can provide synergistic antitumor effect with immunotherapy in lung cancer [5–7]. PTT can induce immunogenic cell death (ICD) of tumor cells, which eliminates the tumor cells by promoting the expression of the calcium reticulin (CRT) on the surface of dying tumor cells as well as the discharge of damage-associated molecular patterns (DAMPs), releasing the “eat me” signals from cancer cells to activates intrinsic immune cells [8, 9]. On the other hand, when tumor cells experience immunogenic cell death, a significant amount of endogenous ATP is also produced, and certain negative regulatory molecules, such ecto-5’-nucleotidase (CD73), are overexpressed on the surface of tumor cells, to rapidly hydrolyze adenosine triphosphate (ATP) to adenosine [10–12]. Adenosine, one of the major metabolisms in tumor, was reported to be overexpressed and correlated with the immune suppression in lung cancer in lung cancer [13, 14]. The elicited adenosine greatly hinders maturation of dendritic cells and leads to an immunosuppressive tumor microenvironment with an abundance of regulatory T cells (Tregs) which hamper the immune response. Preclinical experiments have shown CD73 blockage to be a promising target for immune modulation because it prevents T-cell inactivation and enhances antitumor response by inhibiting adenosine synthesis [15, 16].

The development of nanotechnology has shed light on the improvement of PTT performance, among which black phosphorus (BP) has drawn much of attention since to its potential benefits in biomedical applications [17–20]. The constituent element phosphorus is an important element in living organisms and has a natural biocompatibility superior to other nanomaterials. It is also degradable and eventually degrades to phosphate ions in living organisms to be absorbed by the human body. Meantime, nano-black phosphorus has an extensive specific surface area that makes it suitable for drug molecules loading through physical adsorption or chemical binding, as well as the excellent photothermal conversion capacity for the photothermal treatment of tumor tissues [21–26].

Drug delivery by nanosystem plays important role in the promotion of drug accumulation and treatment efficacy [27–30]. As a naturally occurring polysaccharide with good biologic compatibility, biodegradability, and physiochemical features, chitosan (CS) has already been extensively investigated within the pharmaceutical and drug delivery field [31, 32]. The cross-linking of chitosan can form nanogel which is suitable for drug loading with enhanced penetration ability and controlled release behavior. However, the clearance and safety of chitosan nanoparticles in the vasculature system are still issues need to be considered due to the abundant amino group and positive charge in the surface [33–35]. Biomimetic camouflage with cell membrane, e.g. dendritic cells, cancer cells, and red blood cells, can provide better solution to improve the bioavailability of nanomedicines [36–39]. Previous researches revealed that the coating of erythrocyte membrane (EM) exhibits advantage to the exogenous nanoparticles, such as low immunogenicity, reduced phagocytic clearance, prolonged circulation time, and increased biosafety [40–42]. Due to the easy availability and simple preparing process, the erythrocyte membrane is widely applied in the camouflage of nanoparticles and drug delivery. Furthermore, modification of membrane with high affinitive and specific aptamers, could further enhance the targeting property of nanomedicine [43–48].

**Fig. 1:**
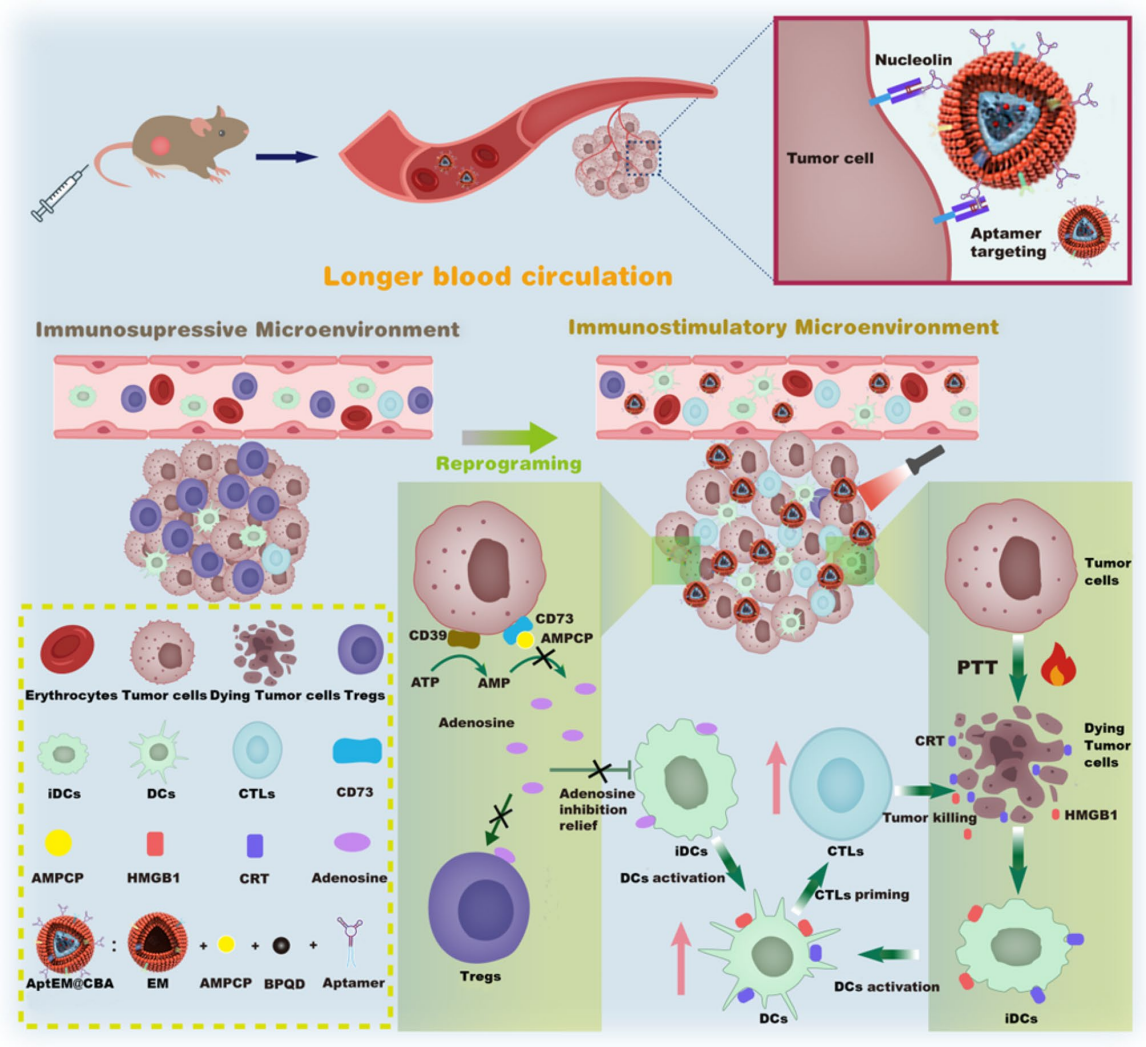
Scheme illustration of biomimetic nanodrug synergizing CD73 blockade and photothermal therapy to modulate tumor environment and boost antitumor immunity

In this work, we constructed a novel biomimetic photothermal nanodrug compositing with CD73 inhibitor α, β-methylene adenosine 5’diphosphate (AMPCP), black phosphorus quantum dots (BPQDs), chitosan nanogel, and aptamer modified erythrocyte membrane, which facilitates the adenosine inhibition of AMPCP to synergize with the photothermal effect of BPQDs to manipulate the tumor immune microenvironment, thus enhancing the subsequent immunotherapy of lung tumors. In this system, the positively charged chitosan adsorbed negatively charged BPQDs and AMPCP through the cross-linking of tripolyphosphate (TPP) to form uniform nanogels, which were then co-extruded with aptamer AS1411-functionalized erythrocyte cell membrane nanovesicles, to obtain the erythrocyte membrane-camouflaged photothermal nanodrug AptEM@CBA. *Via* the active targeting induced by AS1411 aptamer and the long blood circulation time prolonged by erythrocyte membrane, selective enrichment of the nanodrug in tumor sites was achieved. Under irradiation of near-infrared light, the photothermal effect of BPQDs promoted the maturation of dendritic cells, concurrently released AMPCP to suppress the adenosine generation by CD73 blockade which alleviated the impairment of adenosine to dendritic cells and suppressed regulatory T cells, leading to a synergistic boost in the activity of T lymphocytes. *Via* the combination of CD73 blockade with PTT, not only the growth of primary tumors was inhibited, but also the distal tumors were suppressed due to the potential boost of antitumor immunity. This work may shed light on the application of nanodrug in immune modulation based on the adenosine regulation and photothermal treatment, and provides new strategy for the therapy of lung cancer (Fig. 1).

## Materials and methods

### Materials

All the chemicals were used as-received without further purification if not specified otherwise. The bulk black phosphorus (BP) was purchased from Nanjing XFNANO Technology Co., Ltd. 1-Methyl-2-pyrrolidinone (NMP) was bought from Aladdin. CD73 inhibitor AMPCP was purchased from Merck BioInc. Chitosan was purchased from Sigma-Aldrich, Sodium tripolyphosphate (TPP) was purchased from Wokai Biotechnology Co., Ltd, sodium hydroxide was purchased from Sinopharm Chemical Reagent Co., Ltd, and the nucleic acid aptamers AS1411, Lib, 5’Cy5-labeled AS1411 and Lib were all synthesized by Shanghai Biotechnology Co. DSPE-PEG_2000_-maleamide was purchased from Shanghai Pengsheng Biotechnology Co. Cy_5_-NHS was purchased from Changsha Hanchen Biotechnology Co. Elisa assay kits of TNF-a, IFN-γ, and IL-2 were purchased from Beyotime, ELISA Kit for adenosine, BUN, CR, ALT, AST detection was purchased from Shanghai Enzyme-linked Biotechnology Co. Primary antibody of Calreticulin(CRT) and High mobility group protein B1(HMGB1) was purchased from Abcam. Cy3-labeled secondary antibody for CRT was purchased from Boster Biological Technology Co., Ltd and Alexa Fluor® 488-labeled secondary antibody for HMGB1 was purchased from Abcam. Ki67, MMP9, CD8, CD86, FOXP3, CD11b, CD206 and iNOS antibodies used for immunohistochemistry and immunofluorescence were purchased from Abcam. For flow cytometry testing, Antibodies of FOXP3-PE, CD8-PerCP-Cy5, CD4-APC, CD45-APC-Cy7, CD25-BV421, CD3-BV510, MHCII-PE, CD11C-PE-Cy7, CD80-APC, CD86-BV421 were purchased from BD Biosciences. Granzyme B-PE- CY7 was purchased from Thermo.

### Preparation of BPQDs

According to our previous work [49], 25 mg of bulk BP was ground into powder in a mortar and pestle, 25 mL of NMP was added and conducted probe ultrasound at 1200 W (ultrasonic frequency: 19–25 kHz, On / Off cycle: 2 s / 2 s) in an ice bath for 4 h. Then the sample was ultrasonicated at 800 W for 12 h in an ice bath. The supernatant was centrifuged at 7500 ×g for 5 min and the drawn supernatant was centrifuged at 12,000 ×g for 5 min, then the aspirated supernatant was centrifuged for 30 min at 21,000 ×g, the precipitates were obtained as BPQDs.

### Preparation of chitosan nanogel and encapsulation of BPQDs and AMPCP

The preparation of chitosan nanogel and encapsulation BPQDs and AMPCP was similar with the reported method [50–52]. Briefly, under rapid stirring, a certain amount of 1 M aqueous sodium hydroxide solution was added to 2 mg/mL chitosan solution, adjusting the pH to 5–6, then TPP (0.4 mg/mL) was dropwise added and the mixture was continuously stirred for 30 min at room temperature. The mixture was centrifuged 10,000 ×g for 10 min, discarding the supernatant, and resuspending the precipitate with ddH_2_O to obtain the chitosan nanogel solution.

To encapsulate BPQDs and AMPCP in the chitosan nanogel, BPQDs (20 µg/mL), and AMPCP (200 µg/mL) were added together with TPP during the cross-linking process. Following similar subsequent process, the chitosan nanogel containing BPQDs and AMPCP was obtained, and was defined as CBA.

### Preparation of EM@CBA

Red blood red cells were separated from fresh whole blood obtained from C57 mice, washed twice with DPBS, followed by hypotonic treatment with 0.25× DPBS under ice bath conditions for 1 h and then centrifugated at 12,000 rpm for 10 min. The supernatant was discarded, and the residue was washed twice with DPBS to obtain red blood red cell membranes. Then the erythrocyte cell membrane nanovesicles (EM) were prepared by sequentially passing the polycarbonate membrane with 800 nm and 400 nm pore sizes. Then equal amounts of EM were mixed with CB or CBA solution and co-extruded 15 times through a 400 nm pore size polycarbonate membrane to obtain EM@CBA.

### Preparation of AptEM@CBA

3’-SH modified AS1411 nucleic acid aptamer was mixed with an equal amount of DSPE-PEG-Mal, rotary shaking at room temperature for 2 h. Then the mixture was mixed with EM@CBA, and left in an incubator at 37 ^o^C for 30 min. Afterwards, the mixture was centrifuged at 10,000 ×g for 10 min. Then the supernatant was discarded, and the precipitate was resuspended with ddH_2_O to obtain AptEM@CBA.

### Characterization

The TEM images were acquired on the Tecnai G2 F20 S-Twin transmission electron microscope at an acceleration voltage of 200 kV. UV-NIR absorption spectra were acquired at room temperature on a T2601 Ultraviolet-visible Spectrophotometer (Yoke Instruments) with QS-grade quartz cuvettes. The zeta potentials and dynamic diameter were measured on a NanoBrook 90Plus analyzer (Brookhaven Instruments).

### Performance of AptEM@CBA for NIR photothermal conversion

100 µL of different concentrations of AptEM@CBA were added into 96-well plates, and the nanodrug was irradiated with NIR at 808 nm with a power of 1 W cm^− 2^ while the temperature was recorded at specific time points using an infrared thermography camera. For the evaluation of photothermal stability, the nanodrug with BPQDs concentration of 60 µg/mL was irradiated for 5 min with NIR light of 808 nm at a power of 1 W cm^− 2^, and was allowed to cool naturally to room temperature, which was defined as one cycle. 5 cycles were repeated and the temperature data was recorded at specific time points.

### AMPCP loading capacity

CBA and EM were co-extruded through a 400 nm pore size polycarbonate membrane. The extrudate was subjected to ultrafiltration (MWCO 10 KDa, Millipore) and the filtrate was collected. The concentration of the AMPCP in the filtrate was calculated by the UV-Vis-NIR absorption curve of the filtrate. The encapsulation efficiency (EE) of the AMPCP was defined as follows:

Where Ci denoted the initial AMPCP concentration of the mixed system, and Cf denoted the concentration of the filtrate.

### In vitro stability of AptEM@CBA

Equal amount of AptEM@CBA was resuspended with DPBS and DMEM medium respectively with BPQDs concentration of 60 µg/mL, and stored at 4 ^o^C for 7 days. The particle size of the nanodrug was detected and recorded the temperature rise of the nanodrug after irradiated with NIR at a power of 1 W cm^− 2^ for 5 min every other day to assess its photothermal ability.

### Cell culture

The cells used in this experiment, such as LLC (Mouse Lewis lung cancer cells), HACAT (Human immortalized fibroblasts), 293T (Human embryonic kidney cells), and LO2 (Human normal hepatocytes), were all obtained from the cell bank of Chinese Academy of Sciences. LLC were cultured in DMEM basic medium (Gibco, USA) supplemented with 10% FBS (VivaCell, China) and 100 UI mL^− 1^ penicillin and 100 UI mL^− 1^ streptomycin (Solarbio, China). HACAT, 293T, LO2 were cultured in RPMI 1640 basic medium (Gibco, USA) with 10% FBS and 100 UI mL^− 1^ penicillin and 100 UI mL^− 1^ streptomycin. All cells were cultured in a humidified incubator (Thermo Fisher 311 Direct Heat CO_2_ Incubator) at 37 ^o^C and 5% CO_2_.

### Red blood cell membrane protein imprinting

Red blood cell membranes from fresh mouse red blood cells, EM@CBA and AptEM@CBA, were lysed with 2% sodium dodecyl sulfate (SDS) solution and then loading buffer was added. After the performed sodium dodecyl sulfate polyacrylamide gel electrophoresis (SDS-PAGE), coomassie brilliant blue staining was carried out for 2 H, and photograph was taken after elution.

### Aptamer cell targeting and flow cytometry

The 5’-Cy5-labeled AS1411 with the sequence of 5’GGTGGTGGTGGGTTGTGGTGGTGGTGGGTGGG was synthesized by Shanghai Bioengineering Company. To verify the targeting specificity, the 5’-Cy5-labeled Lib with the sequence of 5’ATCCGTTACTCAGAACTAGCNNNNNNNNNNNNNNNATTCGACTCATACATGCATT was also synthesize. Briefly, LLC, 293T, HACAT were digested down with non-enzyme cell detach solution, washed twice with DPBS, and the supernatant was discarded. Then 5’Cy5-labeled AS1411 and Lib aptamer dissolved in binding buffer at a concentration of 300 nM were mixed with the above cell pellets respectively. The mixtures were incubated at 37 ^o^C away from light for 30 min, then washed with washing buffer twice and conducted with flow cytometry by DxP Athena V5-B5-R3 Flow cytometer. The data were processed and analyzed by FlowJo V10.0.7 software.

### In vitro cellular targeting of AptEM@CBA

Cy5-AptEM@CBA and Cy5-LibEM@CBA were obtained by modifying EM@CBA with Cy5-labelled AS1411 and Lib at a concentration of 300 nM, respectively. LLC, 293T, HACAT were digested with trypsin-free digest, then the three cell pellets were resuspended with Cy5-AptEM@CBA and Cy5-LibEM@CBA, respectively, and washed twice with washing buffer after 30 min in an ice bath protected from light, and then analysed by flow cytometry.

### In vitro cellular uptake of AptEM@CBA

Chitosan was co-mixed with TPP, BP, AMPCP, Cy5 and then co-extruded with EM to obtain EM@CBA-Cy5. EM@CBA-Cy5 was modified with DSPE-PEG_2000_-AS1411 to obtain AptEM@CBA-Cy5. LLC, 293T, HACAT cells were inoculated in 96-well plates (3 × 10^4^ cells/well) separately and cultured overnight, then co-incubated with AptEM@CBA-Cy5 for 6 h respectively. Afterwards, the medium was discarded and the wells was washed twice with DPBS. Then the nuclei were stained with 50 μm Hoechst for 15 min, and the fluorescence was observed by inverted fluorescence microscope (Nikon DIAPHOT300, Japan).

### In vitro cytotoxicity

LLC and LO2 cells were inoculated in 96-well plates (1 × 10^4^ cells/well), cultured overnight, and then co-incubated with different concentrations of AptEM@CBA for 12 h. Then MTT reagent was added to detect cell activity. The cell viability was calculated by using the following formula: Cell viability (%) = (Abs sample/Abs control) ×100%. Where Abs sample denotes the absorbance of the solution for the nanodrug treatment group, and Abs control denotes the absorbance of the solution for the normal cultured cells.

### Hemolysis assay

100 µL of whole blood extracted from C57 mice was centrifuged at 400 ×g for 5 min at 4 ^o^C, then the supernatant was discarded and the precipitates were washed twice with DPBS to isolate the blood cells. Then the blood cell clumps were resuspended with DBPS, H_2_O, different concentrations of CBA and AptEM@CBA. After incubated at 37 ^o^C for 6 h, the mixture was centrifuged at 600 ×g for 5 min. To exclude the effect of nanodrug, the supernatant was further centrifuged at 12,000 ×g for 10 min. Then an identical amount of supernatant was pipetted out to a 96-well plate to measure the absorption at 540 nm.

### In vitro calcein AM/PI live-dead staining

LLC cells were inoculated in 96-well plates (3 × 10^4^ cells/well), cultured overnight, and then co-incubated with different concentrations of AptEM@CBA for 2 h. Then the cells were irradiated with NIR at 808 nm for 5 min at 1 W cm^− 2^ and the incubation was continued for 6 h. After the medium was aspirated, and Calcein AM/PI staining was carried out according to the operation manual. Fluorescence was observed using an inverted fluorescence microscope (Nikon DIAPHOT300, Japan).

### In vitro photothermal induced immunogenic cell death (ICD) of tumor cells

LLC cells were inoculated in 96-well plates (3 × 10^4^ cells/well), cultured overnight, and then co-incubated with AptEM@CB(60 µg/mL BPQDs), EM@CBA (60 µg/mL BPQDs, 10 µg/mL AMPCP), AptEM@CBA (60 µg/mL BPQDs, 10 µg/mL AMPCP) separately for 2 h, followed by irradiation with 808 nm near infrared light at 1 W cm^− 2^ for 5 min. For Calreticulin (CRT) detection: After continuous incubation for 6 h, the medium was aspirated and washed cells twice with DPBS, cells were fixed with − 20 ℃ pre-cooled methanol for 10 min and were blocked with 100 µL of blocking solution at room temperature for 2 h. After aspirated out the blocking solution, 100 µL 1:500 dilution of CRT antibody was incubated with the cells at room temperature for 1 h. Then the cells were incubated with 1:200 dilution of Cy3-labeled secondary antibody (BOSTER, China) at room temperature for 1 h. Next, 50 µM Hoechst was used to stain nuclei and fluorescence was observed using an inverted fluorescence microscope. For the detection of high mobility group protein B1 (HMGB1): After continuous incubation for 6 h, the medium was aspirated and washed cells twice with DPBS, 100 µL of 4% paraformaldehyde was used to fix cells which was followed by incubation with 100 µL of blocking solution for 2 h at room temperature. Then cells were incubated with 100 µL of 1:100 dilution of HMGB1 antibody at 4 ℃ overnight. Afterwards, Alexa Fluor® 488-labeled secondary antibody at 1:500 dilution was incubated with cells at room temperature for 2 h. The nucleus was stained with 50 µM Hoechst for 15 min. After washing twice with DPBS, fluorescence was observed using an inverted fluorescence microscope. As for the detection of released ATP: After continuous incubation for 8 h, the medium was collected and centrifuged at 12,000 g for 10 min. The supernatant was retained and ATP levels were measured with an enhanced ATP Assay Kit (Beyotime, USA) according to the instructions.

### In vitro adenosine inhibition assay

LLC cells were inoculated in 96-well plates (3 × 10^4^ cells/well) and cultured overnight, then cells were treated with fresh complete medium, AMPCP (10 µg/mL), AptEM@CB (60 µg/mL BPQDs), AptEM@CB (60 µg/mL BPQDs) + NIR, AptEM@CBA (10 µg/mL AMPCP, 60 µg/mL BPQDs) + NIR, respectively. After irradiation with NIR laser 1.0 W cm^− 2^ for 5 min, the treated cells were continued to be cultured for 8 h. Then the cell culture supernatant was aspirated to be centrifuged at 12,000 ×g for 10 min, and adenosine Elisa assay was performed according to the instructions.

### In vitro facilitation of DCs maturation

Eight-week-old male C57 mice were executed by cervical dislocation, and the limbs were separated with scissors. The muscles of the limbs were stripped, and the femur and tibia were soaked in 75% alcohol for 10 min and then transferred to an ultra-clean bench. A cut was made at each end of the femur and tibia, and the bone marrow cavity was blown and washed with a syringe filled with DPBS. The blowing solution with bone marrow tissue was collected and filtered through a cell sieve with a pore size of 70 μm to obtain a single cell suspension. Then, centrifugation was performed at 400 ×g for 5 min, and the supernatant was discarded. The cell pellet was resuspended with 2 mL erythrocyte lysate, and was left to stand for 3 min. The mixture was centrifuged at 400 ×g for 5 min, and the supernatant was discarded. The cell pellet was resuspended with complete medium of RPMI 1640 supplemented with 10% FBS, 20 ng/mL GM-CSF (PEPROTECH, USA) and 10 ng/mL IL-4 (PEPROTECH, USA) to adjust the cell concentration to 1.5 × 10^6^. The cell suspension was spread into 24-well plates and cultured for 7 days, and the medium was changed every other day to obtain the immature dendritic cells (iDCs). The iDCs were collected and the RPMI 1640 complete medium supplemented with 10% FBS, 20 ng/mL GM-CSF and 10 ng/mL IL-4 was used to adjust the iDCs concentration to 5 × 10^5^/mL, then the iDCs was inoculated into the receiving wells of Transwell 6-well plates. While the LLC cells were spread to the chambers of Transwell 6-well plates and incubated at 37 ℃ for 12 h before different treatments (*n* = 3): DMEM complete medium group, AptEM@CB(60 µg/mL BPQDs) group, AptEM@CB (60 µg/mL BPQDs) + NIR (1.0 W cm^− 2^ for 5 min) group, AptEM@CBA (60 µg/mL BPQDs, 10 µg/mL AMPCP) + NIR group (1.0 W cm^− 2^ for 5 min), and 1 µg/mL lipopolysaccharide( LPS, Solarbio, China) group. Then the chambers were gently placed into the receiving wells and cultured at 37 °C in a cell culture incubator for 24 h. The DCs in the receiving wells were collected and co-incubated with fluorescence labeled antibody against MHCII-PE, CD11C-Pe-Cy7, CD80-APC, CD45-APC-CY7, and CD86-BV421 for flow cytometric analysis.

### Construction of bilateral tumor model

All animal experiments related to this study were approved by the Ethics Committee of the Department of Laboratory Animals, Central South University (CSU-2022-0635). Specific pathogen Free (SPF) grade 4-week-old male C57 mice were purchased from Hunan SJA Laboratory Animal Co., and housed in individually ventilated cages in the barrier environment of the Department of Laboratory Animal Science, Central South University. The mice were ensured access to adequate food and water as well as a certain degree of freedom of movement. The primary tumor was inoculated by subcutaneous injection of 100 µL (2 × 10^6^) of logarithmically growing LLC on the right flank of the mice, and the distal tumor was inoculated by subcutaneous injection of 100 µL l (1 × 10^6^) of LLC cells on the contralateral side of the mice 5 days after inoculation of the primary tumor. Tumor volume was calculated based on the following formula: Tumor volume (mm^3^) = 0.5 × Length × Width^2^.

### In vivo antitumor and distal tumor- inhibitory effects

When the volume of the primary tumor reached ∼ 100 mm^3^, tumor-bearing C57 mice were randomly divided into five groups (*n* = 4), and subjected to different treatments. G1 was the DPBS group, G2 was the AptEM@CBA group, G3 was the AptEM@CB + NIR irradiation group, G4 was the EM@CBA + NIR irradiation group, and G5 was the AptEM@CBA + NIR irradiation group. At 4 h after the intraperitoneal injection of all materials (the amount of BPQDs was 40 µg/mouse, and the amount of AMPCP was 200 µg/mouse), the primary tumors were irradiated by NIR 1 W cm^− 2^ for 10 min. Volume changes of the primary tumor and distal tumor were recorded every other day.

### Flow cytometric analysis of immune cells in tumor tissues

At the end of the treatment, mice were executed and tumor tissues on both sides were quickly stripped. Appropriate size of tumor tissue mass was put into 24-well plates and cut into ∼ 1 mm^3^ pieces with scissors. Then tissue dissociation solution (Absin, China) was added, static at 37 ℃ for 30 min and 2 mL of DPBS-BSA (0.25% BSA) solution was used to terminate the digestion and then 70 μm cell filters were used to obtain the single cell suspension. The single cell suspension was centrifuged at 400 ×g for 5 min in 4 ℃ and the cell pellets were blocked with Mouse BD FC Block followed by antibody incubation. Fluorescence labeled antibodies used to identify T cells and regulatory T(Treg) cells were FOXP3-PE, CD8-PerCP-CY5.5, CD4-APC, CD45-APC-CY7, CD25-BV421, CD3-BV510. Fluorescence labeled antibodies used to characterize DC cells were MHC II-PE, CD11C-Pe-cy7, CD80-APC, CD45-APC-CY7, CD86-BV421. Dxp Athena (Cytek, USA) was used to acquire the flow data which were analyzed by Flowjo-V10 software.

At the end of the treatment, mice were executed and tumor tissues on both sides were quickly stripped. Appropriate size of tumor tissue mass was put into 24-well plates and cut into ∼ 1 mm^3^ pieces with scissors. Then tissue dissociation solution (Absin, China) was added, static at 37 ℃ for 30 min and 2 mL of DPBS-BSA (0.25% BSA) solution was used to terminate the digestion and then 70 μm cell filters were used to obtain the single cell suspension. The single cell suspension was centrifuged at 400 ×g for 5 min in 4 ℃ and the cell pellets were blocked with Mouse BD FC Block followed by antibody incubation. Fluorescence labeled antibodies used to identify T cells and regulatory T(Treg) cells were FOXP3-PE, CD8-PerCP-CY5.5, CD4-APC, CD45-APC-CY7, CD25-BV421, CD3-BV510. Fluorescence labeled antibodies used to characterize DC cells were MHC II-PE, CD11C-Pe-cy7, CD80-APC, CD45-APC-CY7, CD86-BV421. Dxp Athena (Cytek, USA) was used to acquire the flow data which were analyzed by Flowjo-V10 software.

### Detection of serum immunostimulatory cytokines

When the treatment was finished, whole blood of mice was collected into 1.5 mL centrifuge tubes and left to stand at 4 ℃ for 10 min, then centrifuged at 400 ×g for 5 min. The serum was aspirated out, and the levels of immunostimulatory cytokines TNF-ɑ, IFN-γ, and IL-2 in serum were detected by using the ELISA Kit (Beyotime, China) according to the instructions.

### Adenosine level in tumour tissue

At the end of the treatment regimen, an appropriate amount of tumour tissue was excised and cut into small pieces of approximately 1mm3, followed by grinding with a mortar and adding the corresponding amount of PBS (mass-volume ratio of 1:4). The homogenate was collected and then centrifuged at 12,000 g for 10 min at 4 ^o^C, then the supernatant was taken and tested for adenosine content using a adenosine Elisa assay.

### Tumor tissue immunohistochemistry and immunofluorescence staining

T cell marker CD8, mature DC marker CD86, Treg marker FOXP3, MDSC marker CD11b, M1-like TAMs marker iNOS and M2-like TAMs marker CD206 were detected by immunofluorescence. The primary distal tumors were submerged in 4% paraformaldehyde for 1 day, then placed in liquid paraffin, and then sliced into 4 μm thickness sections using a slicer. The slices were treated with 1% Triton X-100 and 30% H_2_O_2_ after baked in an oven at 60 °C for 60 min. Antigen repair was performed with 0.01 M sodium citrate at pH6.0, followed by blocking with 10% goat serum for 30 min. Freshly prepared DAB chromogenic solution was added and then the sections were stained with hematoxylin and sealed with neutral resin for observation. Primary antibodies for CD8, CD86, FOXP3, CD11b, iNOS and CD206 were incubated with sections overnight at 4 °C, and then incubated with fluorescence labeled goat anti-rabbit secondary antibody (Abcam, England) for 1 h at room temperature.

### In vivo biosafety

To monitor the effect of treatment on the health of mice, one day before the end of the treatment regime, whole blood of mice was taken in EDTA-2 K anticoagulation tubes by tails cutting. The levels of White blood cells (WBC), red blood cells (RBC), hemoglobin (HGB) and platelets (PLT) were detected by XN-1000-B1 fully automated hemocyte analyzer (Sysmex, Japan). After the mice were sacrificed, the major organs including heart, liver, spleen, lung and kidney were isolated, fixed in 4% paraformaldehyde for 12 h, paraffin embedded and sectioned. After deparaffinization, the sections were stained with hematoxylin and eosin, then dehydrated and hyaluronized, and sealed with neutral resin. The serum was separated and the levels of Blood urea nitrogen (BUN), creatinine (CR), alanine aminotransferase (ALT), and aspartate aminotransaminase (AST) usually refers to were detected by the corresponding ELISA Kit (Shanghai Enzyme-linked Biotechnology Co, China).

### Statistical analysis

The experimental results were expressed as mean standard deviation (*n* = 3 in cell experiments and *n* = 4 in animal experiments). Two-sided Student’s t-test was used between the two groups of data, and one-way ANOVA and Dunnett’s multiple comparisons test were used to compare the statistical differences between several groups. We considered differences to be statistically significant at the following p-values: *p < 0.05, **p < 0.01, ***p < 0.001 and ****p < 0.0001. All statistical analyses were performed using GraphPad Prism 8.0.1 software.

## Results and discussion

### Preparation and characterization of AptEM@CBA

**Fig. 2:**
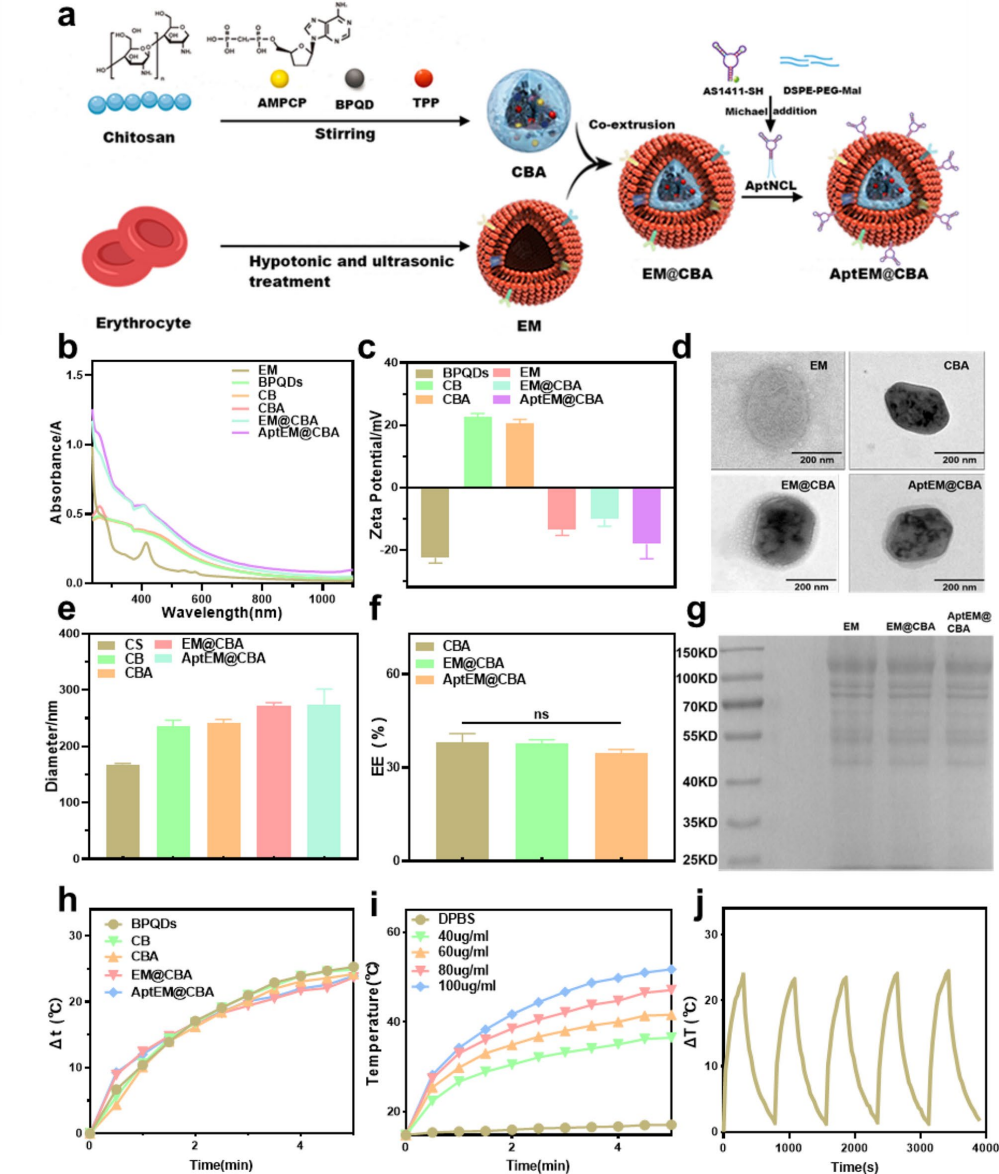
Preparation and characterization of AptEM@CBA. (**a**) Schematic of the synthesis of AptEM@CBA. (**b**) Absorption, (**c**) Zeta Potential, (**d**) TEM images, and (**e**) Diameter of AptEM@CBA. (**f**) Encapsulation efficiency (EE, %) of AMPCP in AptEM@CBA. (**g**) Integrity of membrane proteins of EM in AptEM@CBA. (**h**) The photothermal effect of different materials containing BPQDs. (**i**) Photothermal capacity of AptEM@CBA with different BPQDs concentrations. (**j**) Photothermal stability of AptEM@CBA in five photothermal cycles

The preparation of the biomimetic photothermal nanodrug AptEM@CBA was illustrated in Fig. 2a. α, β-methylene adenosine 5’diphosphate (AMPCP) was employed as CD73 inhibitor, since high intratumoural signal of CD73 was found to be a prognostic factor of overall survival of non-small cell lung cancer (NSCLC) in TCGA cohorts (Figure S1). Black phosphorus quantum dots (BPQDs) were chosen as the photothermal agents and was prepared using the liquid exfoliation method (Figure S2). The biopolymer chitosan was selected as the carrier of AMPCP and BPQDs. Crosslinking of chitosan with TPP formed nanoscale hydrogel particles, encapsulating the hydrophilic BPQDs and AMPCP inside to form chitosan/BPQDs/AMPCP nanocomposites (abbreviated as CBA). Furthermore, the encapsulation was facilitated by the electrostatic interaction between the negative charge of BPQDs and AMPCP and the positive charge of chitosan. On the other hand, the ultrasonic treatment and hypotonic cracking of mice red blood cells produced the erythrocyte membrane (EM). Co-extrusion of CBA and EM over polycarbonate membrane coated the surface of CBA with EM to form EM@CBA. In the final step, the AS1411 aptamer was modified with a long aliphatic chain by Michael addition reaction between the thiol group of AS1411 and DSPE-PEG-Mal, which was further infused into the lipid bilayer structure of EM to form AptEM@CBA.

To confirm the structure of AptEM@CBA, UV-Vis-NIR was first performed, from which we obtained the integration of absorbing band of BPQDs, peak of AMPCP, AS1411 and EM in the absorption spectrum of AptEM@CBA (Fig. 2b). The zeta potential of different particles was also measured. As shown in Fig. 2c, CBA presented positive charge due to the abundant amino group of chitosan. With coating of cell membrane, the potential of EM@CBA was reversed to negative, which was further negated by the infusion of aptamer. Transmission Electron Microscopy (TEM) was further performed to confirm the encapsulation of BPQDs and coating of EM, from which we could obviously observe black dots inside the chitosan nanogel and the membrane structure wrapped around AptEM@CBA (Fig. 2d). The size distribution of the nanoparticles was obtained *via* dynamic light scattering (DLS). As shown in Fig. 2e, the average size of chitosan nanogel was around 160 nm, while embedding of BPQDs and AMPCP increased the size to about 240 nm. With coating of EM, the diameter of EM@CBA was further increased to 270 nm, which was not changed by the insertion of AS1411 in the AptEM@CBA.

To verify the encapsulation efficiency (EE) of AMPCP in the nanodrug, the ratio between the absorbance of the residue and overall AMPCP during the encapsulation process was measured, which calculated the EE to be 34.5%, significantly higher than that without chitosan crosslinking (Fig. 2f and S3). Moreover, to further confirm the wrapping of EM on AptEM@CBA, red blood cell surface membrane proteins were extracted and subjected to SDS-PAGE analysis. As shown in Fig. 2g, the distribution of protein in EM@CBA and AptEM@CBA kept same with EM, suggesting that the coating process hardly affected the integrity of surface membrane proteins in erythrocytes.

The photothermal ability of AptEM@CBA was further tested. As shown in Fig. 2h, after five minutes of 808 nm laser radiation at a power of 1.0 W cm^− 2^, the temperature change of AptEM@CBA, EM@CBA, CBA, and CB kept similar with that of bare BPQDs at the same concentration, indicating that the encapsulation of BPQDs in the nanogel and the wrapping of EM didn’t impair the photothermal performance of BPQDs. Moreover, the photothermal performance was correlated with the BPQDs concentration embedded in AptEM@CBA. With more loading of BPQDs, the higher temperature rise was realized (Fig. 2i). Furthermore, the photothermal stability of AptEM@CBA was tested by repeated irradiation-cool cycles. As demonstrated by Fig. 2j, following five consecutive cycles, the rise of temperature could keep similar with the initial ones, demonstrating good photothermal stability of AptEM@CBA. The stability of nanodrug was further studied by immersing AptEM@CBA in DPBS and DMEM solution for 7 days. As illustrated in Figure S4, the particle size and dispersion index remained almost unchanged, as well as good photothermal capacity, showing good stability of AptEM@CBA.

### In vitro cytotoxicity and tumor targeting of AptEM@CBA

**Fig. 3:**
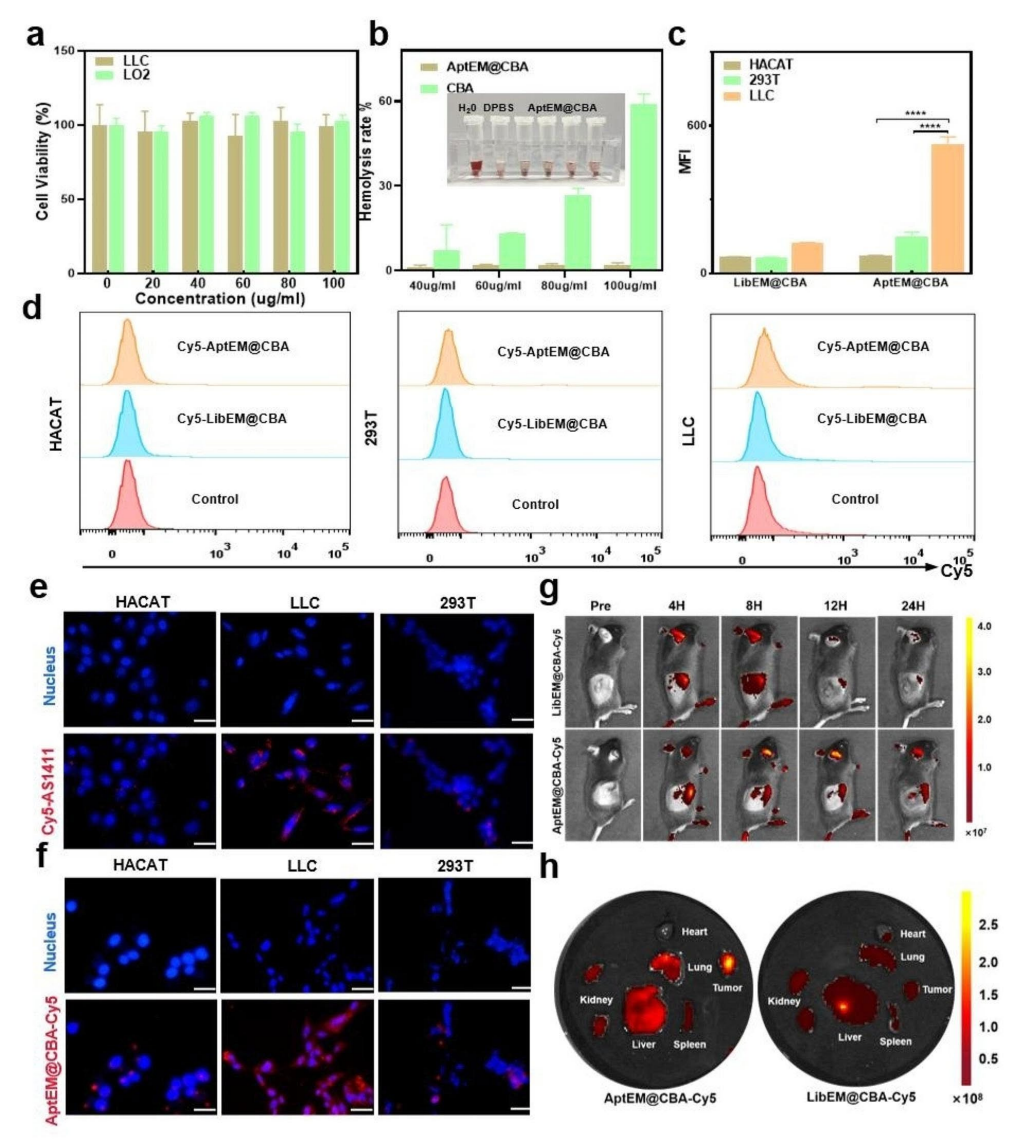
Cytotoxicity and targeting trials of AptEM@CBA in vitro and in vivo. (**a**) Cytotoxicity of AptEM@CBA with different BPQDs concentrations. (**b**) Hemolysis assay of AptEM@CBA, the embedded image is a picture of red blood cells after AptEM@CBA treatment. (**c**) Quantification, and (**d**) flow cytometric analysis of AptEM@CBA targeting HACAT, 293T and LLC cells. (**e**) Inverted fluorescence microscope images of AS1411 targeting HACAT, 293T and LLC cells. (**f**) Phagocytosis of fluorescence labeled AptEM@CBA in HACAT, 293T and LLC cells. Scale bar: 10 μm. (**g**) in vivo distribution, and (**h**) the enrichment at tumor site of the Cy5-labeled AptEM@CBA. *n* = 3. ****p<0.0001

The biosafety of nanomaterials was a prerequisite for their possibility in application. To test the biosafety of the nanodrug, different concentrations of AptEM@CBA were incubated with LLC and LO2 cells for 12 h, and the MTT results showed that AptEM@CBA was non-toxic to both cancer cells and normal human hepatocytes even though the embedding BPQDs was as higher as 100 µg/mL, and the viability rate of cell consistently exceeded 90% (Fig. 3a). What’s more, the erythrocyte hemolysis experiments showed that the bare CBA nanoparticles without EM coating may cause relative higher hemolysis rate due to the strong positive charge on the surface of chitosan, while AptEM@CBA caused rarely hemolysis at the same concentration of embedding BPQDs, showing that EM wrapping rendered AptEM@CBA with improved biocompatibility (Fig. 3b).

Nanodrugs with biologically targeting effects could increase the enrichment of drugs at specific locations, as well as improve the drug utilization to reduce the side effects during the treatment process. We first verified the targeting of aptamer AS1411 to nucleolin, which was highly expressed on the surface of lung cancer cells. Flow cytometric analysis of LLC cells which were incubated with 300 nM Cy5-labeled AS1411 showed that the adsorption of AS1411 to LLC was significantly enhanced comparing with normal HACAT and 293T cells (Figure S5). Similarly, we examined the cellular targeting of Cy5-AptEM@CBA, and the flow cytometric results showed that Cy5-AptEM@CBA had a significantly stronger binding ability to LLC compared to HACAT and 293T cells, suggesting that the AS1411 aptamer modified on the surface of AptEM@CBA was capable to endow the nanoparticle with the targeting ability to lung cancer cells (Fig. 3c-d). Observation with fluorescence microscopy further confirmed the target binding of aptamer to lung cancer cells, where the LLC cells displayed much stronger red fluorescence (Fig. 3e and S6). Subsequently, the internalization of AS1411 modified nanodrug AptEM@CBA-Cy5 in LLC, 293T and HACAT cells were tested separately. A large amount of red fluorescence was observed inside the LLC cells after incubation with AptEM@CBA-Cy5, whereas much less fluorescence was found in the 293T and HACAT cells incubating with AptEM@CBA-Cy5 (Fig. 3f and S7). These results demonstrated that the AS1411 aptamer could significantly promote the targeting ability and phagocytosis of nanodrugs by tumor cells to achieve better treatment.

Based on the above results, the in vivo distribution of AptEM@CBA was further explored. C57 mice were injected with AptEM@CBA-Cy5 and LibEM@CBA-Cy5 respectively *via* the tail vein, and in vivo fluorescence imaging was carried out at specific timepoints following injection. As shown in Fig. 3g, AptEM@CBA-Cy5 achieved obvious enrichment at the tumor site at 8 h post injection, and the fluorescence could still be detectable at 24 h post injection. On the contrary, at the same timepoints of 12 h, the fluorescence signal of LibEM@CBA-Cy5 was significantly reduced and was barely perceptible by 24 h after injection. These results demonstrated that the specific targeting of tumor cells by AS1411 aptamer could enhance the retention time of the nanodrugs at the tumor site, thus resulting in stronger enrichment at the tumor site. In addition, the mice were sacrificed and their main organs extracted 12 h after the injection so that to obtain ex vivo tissue fluorescence photographs. As revealed in Fig. 3h and S8, the AptEM@CBA-Cy5 treated mice exhibited a significantly greater overall red fluorescence in the tumor site compared to the LibEM@CBA-Cy5 mice, further confirming the specific targeting of AptEM@CBA in tumor tissues.

### In vitro photothermal ablation capacity and induction of DCs maturation

**Fig. 4:**
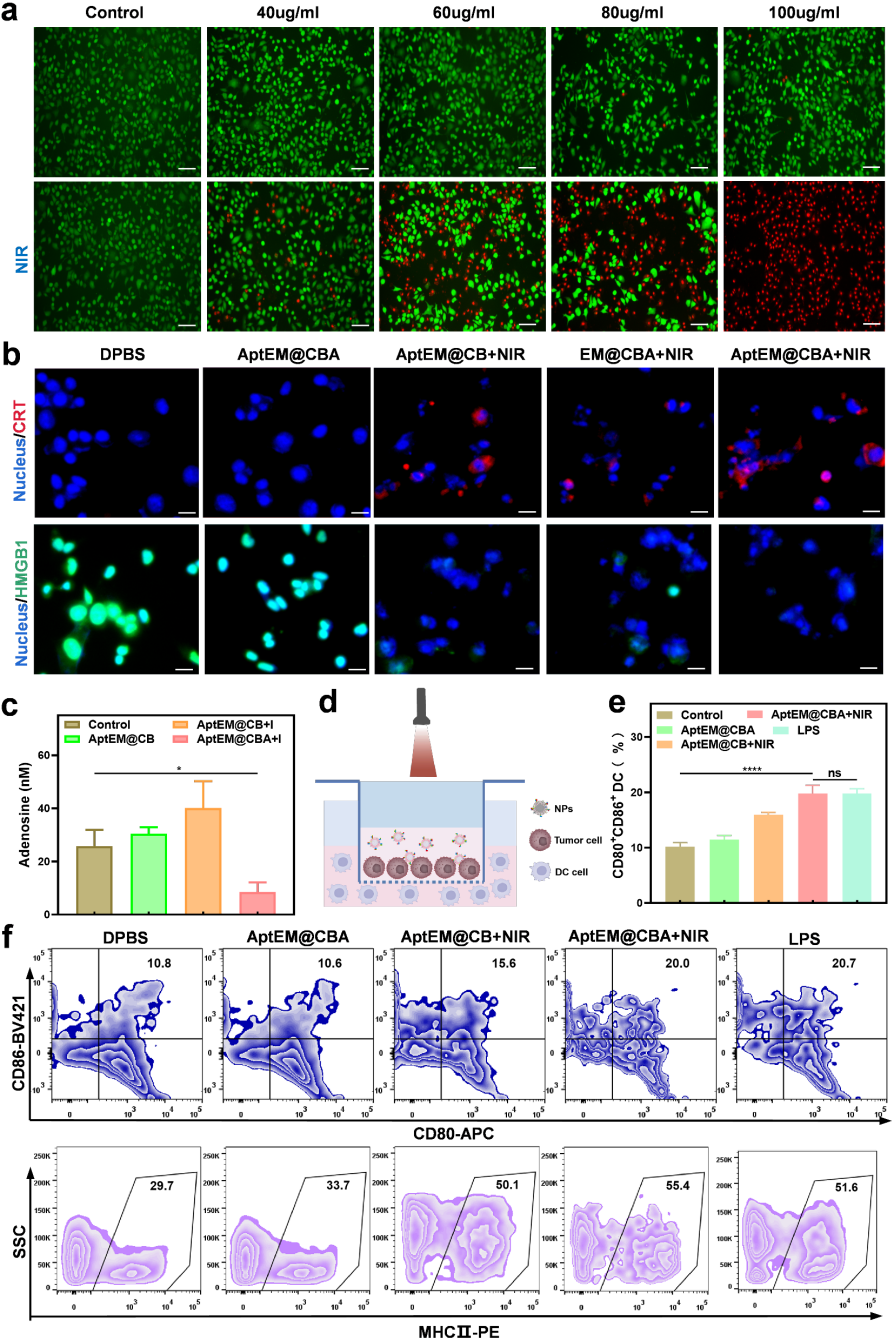
In vitro functional validation of AptEM@CBA. (**a**) Photothermal ablation effect of AptEM@CBA with different BPQDs concentrations. Scale bar: 50 μm. (**b**) The immunofluorescence images of CRT and HMGB1 levels in LLC cells with different treatment. Scale bar: 10 μm. (**c**) Adenosine inhibitory effect of AptEM@CBA on LLC. (**d**) The schematic diagram of AptEM@CBA induced DC maturation after NIR irradiation. (**e**) The quantitative analysis, and (**f**) Flow cytometric detection of DC maturation induction (The gating strategy is shown in Figure S12). *n* = 3. *p<0.05; ****p<0.0001

The death of tumor cell triggered by photothermal treatment will promote the release of damage-associated molecular patterns (DAMPs), such as Calreticulin (CRT), high mobility group protein B-1 (HMGB-1) and ATP, efficiently activating the innate and adaptive anti-tumor immune response and causing the immunogenetic death of tumor cells (ICD). To evaluate the ICD effect of the nanodrug, the i*n vitro* photothermal ablation of tumor cells by AptEM@CBA was firstly evaluated. As shown in Fig. 4a and S9, the photothermal treatment can efficiently ablate the tumor cells in a concentration-dependent manner, whereas no cell death was observed under non-photothermal conditions even the concentration of BPQDs in AptEM@CBA was up to 100 µg/mL. At the next step, we examined the expression of cellular CRT and HMGB1, as well as the release of ATP after photothermal treatment. The results showed that the expression of CRT was increased with the temperature escalation, while the content of HMGB-1 in the nucleus gradually was decreased, and the amount of ATP in the cell culture medium increased, confirming the ICD effect generated by the photothermal irradiation with AptEM@CBA (Fig. 4b and S10).

The generation of adenosine by CD-73 induced decomposition of ATP accompanying with PTT impair the immune response in negative feedback, hence we studied the inhibition effect of AptEM@CBA on adenosine production. As displayed in Fig. 4d, comparing with the control group, the adenosine content in the culture supernatant of LLC cells treated with AptEM@CB which contains no AMPCP was increased under laser irradiation. On the contrary, treatment with AptEM@CBA containing 20 µg/mL of AMPCP significantly decreased the adenosine level under the same photothermal conditions (*p* < 0.05), demonstrating that AMPCP in AptEM@CBA could exert an inhibitory function on adenosine production (Fig. 4c).

Combining the ICD effects and alleviation of adenosine, the synergistic activation of dendritic cells was expected. To confirm this assumption, bone marrow cells were extracted from 8 weeks old C57 mice and induced differentiation to acquire the immature dendritic cells (iDCs). Then the iDCs were cultured in the lower chamber of Transwell plates, with LLC cells and AptEM@CBA (containing 60 µg/mL of BPQDs and 20 µg/mL of AMPCP) in the upper channel. The upper channel was irradiated with 808 nm laser upon power of 1.0 W cm^− 2^ for 5 min for stimulating the maturation of iDCs (Fig. 4d). At 1 day after irradiation, the DCs cells in the lower chamber was collected, and stained for flow cytometric analysis. According to Fig. 4e-f, LLC cells treated with AptEM@CBA didn’t significantly affect the iDC maturation, but the photothermal irradiation with AptEM@CB could cause an increased ratio of iDC maturating to CD86^+^CD80^+^ DCs (16%), which was higher than that of the untreated group’s (10.17%). Moreover, irradiation with AptEM@CBA which containing the AMPCP, could further facilitate the maturation of iDCs higher to 20.0%. Similar results could be seen in the expression of MHCII (Fig. 4f and S11). Accordingly, these results demonstrated that CD73 blockade can synergistically initiate immune responses of photothermal-triggered ICD effect.

### In vivo anti-tumor effect of AptEM@CBA

**Fig. 5:**
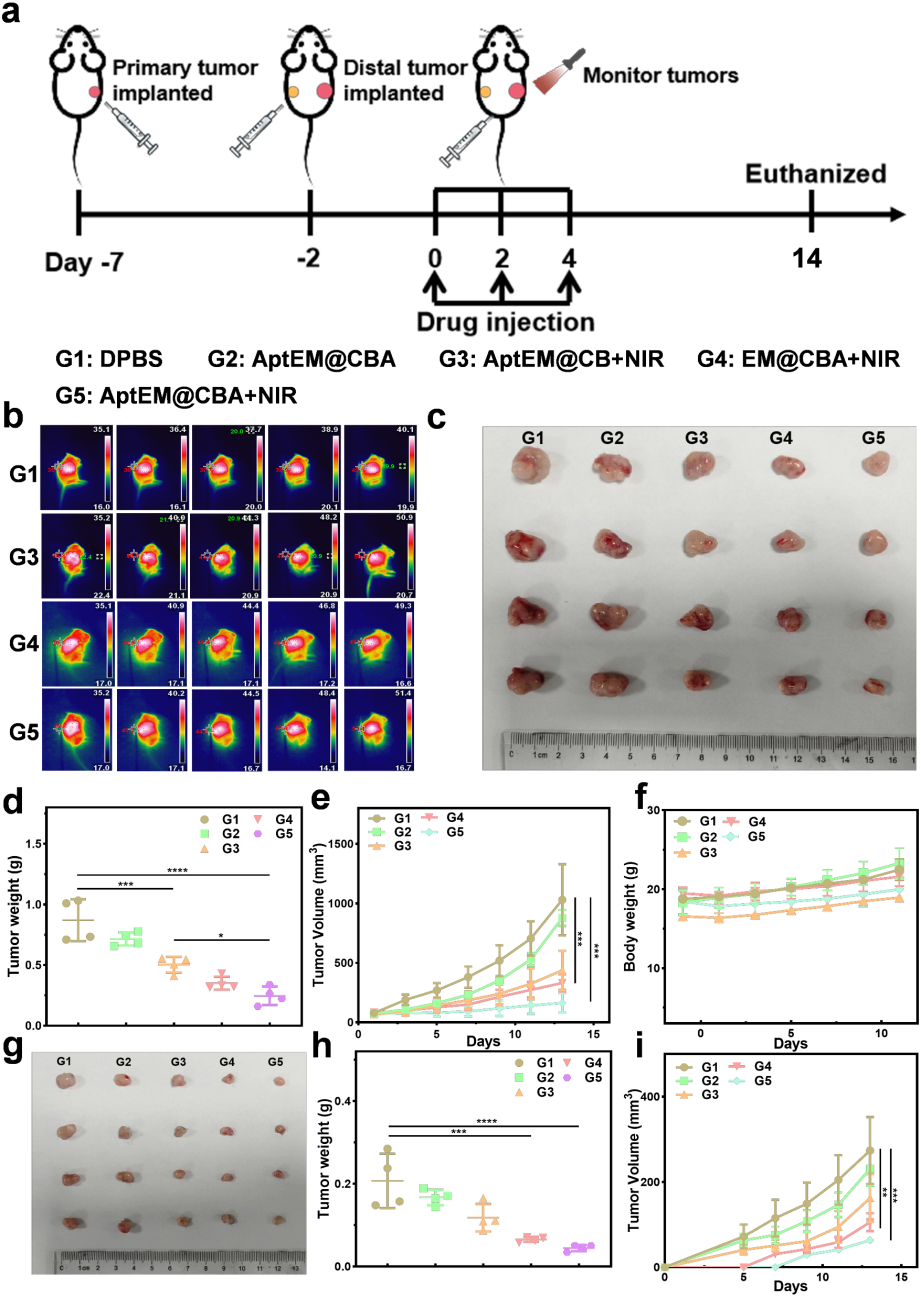
Anti-tumor therapeutic effect of AptEM@CBA on tumor-bearing C57 mice. (**a**) Schematic diagram of the treatment protocol. (**b**) Infrared thermographic maps of the tumor sites under NIR irradiation. (**c**) Images of the main tumors that were collected from different mouse groups after therapy. (**d**) Mass of collected primary tumors. Curves of (**e**) primary tumor growth, and (**f**) body weight in mice with different treatment. (**g**) Pictures of distal tumors collected from different groups of mice at the end of the treatment. (**h**) the mass of the extracted distal tumors in different groups. (**i**) Volume change curves of the distal tumor during the treatment. *n* = 4. *p<0.05; **p<0.01; ***p<0.001; ****p<0.0001

Based on the good targeting, potential photothermal ability and immune initiation effects of AptEM@CBA, we further explored the anti-tumor efficacy in tumor-bearing mice as illustrated in Fig. 5a. A primary LLC tumor was first implanted in the right lateral hind hip in C57 mice, followed five days later by an additional distant tumor in the left lateral hind hip. After the volume of the primary tumor grew up to 100 mm^3^, five different treatments were conducted on the primary tumor as follows: DPBS (G1), AptEM@CBA (G2), AptEM@CB plus NIR irradiation (G3), EM@CBA plus NIR (G4) and AptEM@CBA plus NIR (G5). The volume of the primary and distal tumors of the mice were recorded together with the body weights every other day.

An infrared thermography camera was used to record the temperature changes at the tumor sites upon NIR irradiation during the treatment process. As shown in Fig. 5b, treatment with AptEM@CB, EM@CBA and AptEM@CBA were able to elevate the localized temperatures after NIR irradiation compared with DPBS. Moreover, AptEM@CBA and AptEM@CB raised the temperature up to 51.4 and 50.9 °C respectively, even higher than EM@CBA group (49.3 °C), which might be attributed to the active targeting and enhanced enrichment of nanodrug in tumor sites induced by AS1411 aptamer.

In order to gain better therapeutic effect, three repeated drug injection and photothermal irradiation were performed. After ten days of further feeding, the mice were sacrificed and the tumors were harnessed. As shown in Fig. 5c-e, treatment of AptEM@CBA alone could weakly suppress the grow of tumor, which might be attributed to the adenosine inhibition by CD73 blockade *via* AMPCP. NIR irradiation with AptEM@CB could inhibit tumor more efficiently, indicating the photothermal ablation of tumor. The tumor volume was also reduced by treatment of EM@CBA plus irradiation, implying the synergistic suppression of tumor by combination of photothermia and CD73 blockade. The tumor suppression efficiency reached highest for the AptEM@CBA + NIR group, indicating that AS1411 aptamer induced higher PTT efficiency and CD73 blocking with more accumulation of nanodrug in tumors. What’s more, all these treatments didn’t impair the tumor weight (Fig. 5f). These results demonstrated that CD73 inhibition combined with photothermal treatment could provoke stronger anti-tumor effects without harming the health of mice.

Ki67 is known as an important marker of tumoral malignant proliferation, and MMP9 is intimately associated with tumor invasion. AptEM@CBA + NIR significantly lowered the expression of MMP9 and Ki67 in tumor tissues, according to the immunohistochemistry results of tumor sections, further confirming the potential inhibition on tumor growth and invasion (Figure S13). Moreover, the distal tumor volume change was also recorded at the end of treatment. Interestingly, the distal tumors of the mice in the AptEM@CBA + NIR group exhibited the smallest tumor volume comparing with all other groups (Fig. 5g-i), illustrating the robust distal tumor inhibition effect rendered by AptEM@CBA + NIR. It was also found that the inhibition of distal tumors of AptEM@CB + NIR group was not significant, even though it could efficiently inhibit primary tumors. The powerful distal tumor inhibition by AptEM@CBA + NIR may be attributed to the effective systemic anti-tumor immune response activated by the combination of photothermal irradiation and CD73 inhibition.

### Antitumor immunoregulatory mechanism of AptEM@CBA

**Fig. 6:**
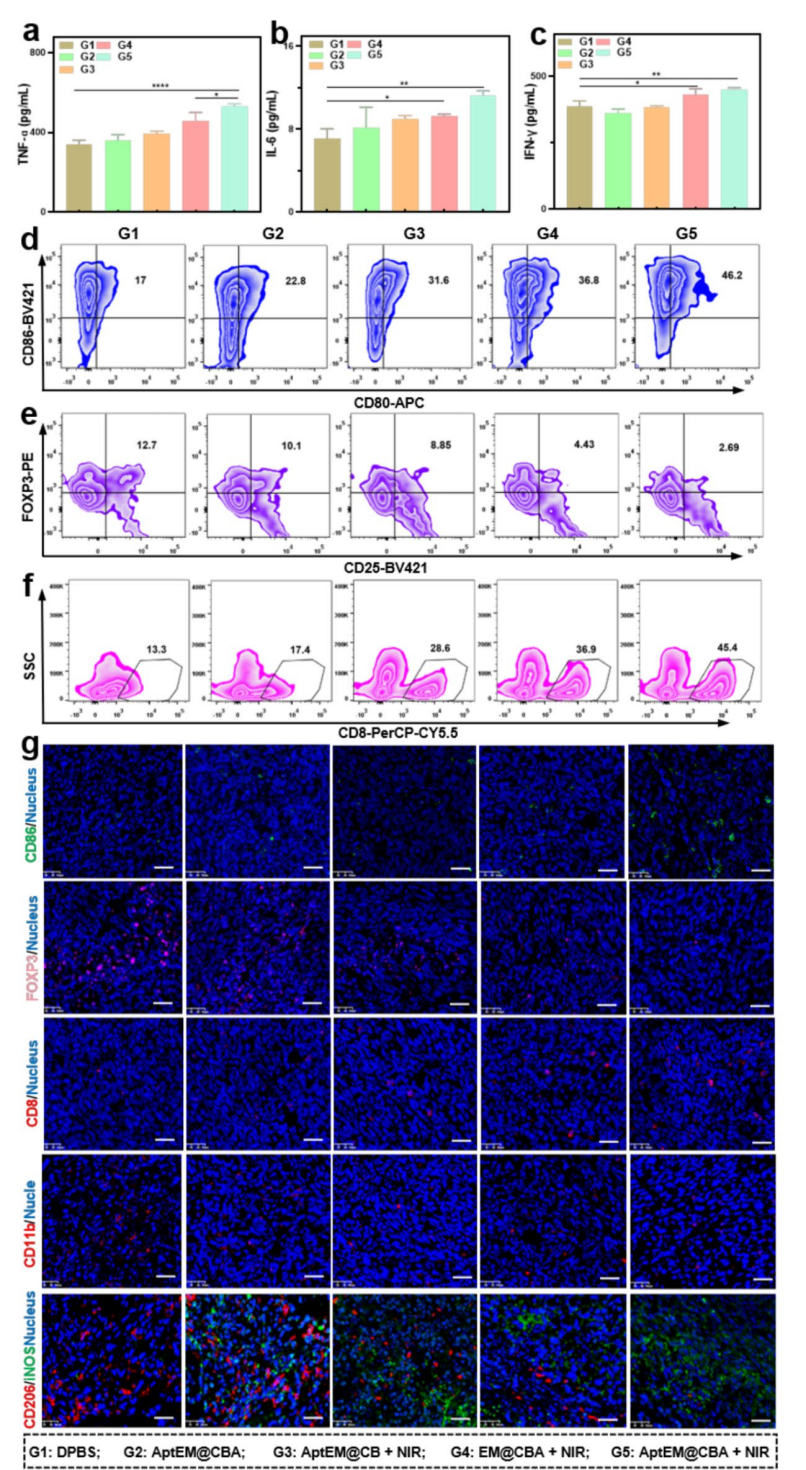
Immunomodulatory effects of AptEM@CBA. Levels of immunostimulatory cytokines (**a**) TNF-α, (**b**) IL-6, and (**c**) IFN-γ in serum of mice from different groups after the treatment. Representative plots of flow cytometric analysis of (**d**) CD80^+^CD86^+^ DCs. (**e**) CD25^+^FOXP3^+^ Tregs, and (**f**) CD8^+^ T cells. (The gating strategy is shown in Figure S17 and S18). (**g**) Representative immunofluorescence images of CD86^+^ DCs, FOXP3^+^ Tregs, CD8^+^ T cells, CD11b^+^MDSCs and iNOS^+^M1-type/CD206^+^ M2-type TAMs in the primary tumor tissues. Scale bar: 50 μm. *n* = 4. *p<0.05; **p<0.01; ****p<0.0001

To better understand the mechanism of potent suppression of primary and distal tumor by AptEM@CBA, immunomodulation of the organism triggered by the treatment was further explored. The adenosine level of the tumour tissue was first examined. As demonstrated in the Figure S14, photothermal treatment with AptEM@CB increased the level of adenosine in the tumour tissue to a certain extent. However, the involvement of AMPCP in the photothermal treatment could significantly inhibit the rise of adenosine, as illustrated in the EM@CBA + NIR and AptEM@CBA + NIR groups. Moreover, the inhibitory effect of AptEM@CBA was even higher than that of EM@CBA, which may be speculated to be the enhanced accumulation of the nanodrug at the tumour site induced by AS1411 aptamer. The serum levels of immunostimulatory cytokines such as IFN-γ, TNF-α, and IL-6 which are inflammation signals essential for boosting systemic immunity were also estimated. TNF-α and IL-6 can prime the activation of B lymphocytes and T cells, and IFN-γ can effectively enable the activation of the antigen-presenting cells. As shown in Fig. 6a-c, the AptEM@CBA + NIR group had significantly higher levels of TNF-α, IL-6, and IFN-γ than the other groups, according to the results of the Elisa test, indicating the increase of inflammation signal in serum promoted by combination of CD73 blockade and photothermia for systemic immunity stimulation.

Activated DCs cells are the predominant antigen-presenting cells essential for initiating antitumor immunity. The maturation of DCs in tumor locations was investigated by utilizing flow cytometry. As shown in Fig. 6d and S15a, AptEM@CBA alone could increase the proportion of CD80^+^CD86^+^DCs compared with the DPBS group, indicating AMPCP could promote the activation of DCs in a certain level. On the other hand, the involvement of photothermia could also promote the ratio of CD80^+^CD86^+^DCs as presented in the AptEM@CB + NIR group. In addition, incorporation of AMPCP with photothermia AptEM@CBA + NIR group could further promote the proportion of CD80^+^CD86^+^DCs which was much stronger than the PTT alone (36.7% Vs 28.9%), highlighting the promotion effect of CD73 blockade combining with photothemia to DCs maturation.

Regulatory T cells (Tregs) are the primary immunosuppressive cells that sustain the tumor’s immune cell-suppressive milieu by preventing effector T cell infiltration and DC activation. The inhibitory effect of AptEM@CBA towards Tregs was also analyzed. As shown in Fig. 6e and S15b, the proportion of CD25^+^FOXP3^+^ Tregs in tumor tissues of the DPBS group 9.8%, whereas the proportion of Tregs treated with AptEM@CB + NIR was reduced to 6.8%, which was not significantly different from that in group DPBS. Interestingly, treatment of AptEM@CBA + NIR could further reduce the proportion of Tregs to 2.2% indicating the CD73 blockade could enhance the inhibition of Tregs’ immunosuppression with photothermia (*p* < 0.01).

With maturation of DCs and suppression of Tregs, the T cells involved in the recognition and attacking of tumor cells were significantly revived. As illustrated in Fig. 6f and S15c, the proportion of CD8^+^T cells in the DPBS group was only 16.1%, which demonstrated the antitumor immunity was sturdy suppressed in LLC solid tumors. Treatment of AptEM@CBA or photothermia alone could increase the proportion of CD8^+^T cells to 22.8% and 32.7% respectively. Remarkably, the CD8 + T cells in the AptEM@CBA + NIR group could be significantly elevated to 41.3%), which was significantly higher than that in the EM@CBA + NIR and AptEM@CB + NIR group individually, exhibiting effective stimulation of antitumor immune response. We similarly analyzed the changes in the proportion of CD4^+^T cells, founding that treatment of AptEM@CBA + NIR could increase the proportion of CD4^+^ T cells with significance (*p* < 0.05, Figure S15d-e).

Immunofluorescence staining study of CD86^+^DCs, FOXP3^+^Tregs, CD8^+^ T cells, CD11b^+^ Myeloid-derived suppressor cells (MDSCs) and tumor associated macrophages (TAMs) in tumor tissue sections was performed to further confirm the enhanced immune response. As displayed in Fig. 6g, the fluorescence signal of CD86 was remarkable increase after treatment of AptEM@CBA plus NIR irradiation, while the florescence of FOXP3 was significantly decreased, resulting in the enhancement CD8 fluorescence. Furthermore, CD11b^+^ MDSCs and M2-type TAMs exerting immunosuppressive effects in tumour tissues were also measured. The immunofluorescence results showed that AptEM@CBA + NIR was able to inhibit CD11b^+^ MDSC and CD206^+^ M2-type TAMs while promoting the level of iNOS^+^ M1-type TAMs compared with other treatment groups (Fig. 6g). Collectively, these results above indicated that the incorporation of CD73 blockade with photothermal therapy could substantially boost the maturation of DCs, restrain the activity of Tregs and MDSCs, and promote the immune T-cells and M1-type TAMs activation, thus reversing the immunosuppressive tumor microenvironment and establishing the groundwork for efficient primary tumor clearance and distal tumor suppression.

Based on the potent distal tumour suppression effect of AptEM@CBA + NIR, we performed immunofluorescence analysis for immune cells in distal tumor tissue sections. As shown in Fig. 16, the immunofluorescence results indicated that the levels of immunostimulatory cells in the distal tumors were significantly increased with treatment of AptEM@CBA + NIR, such as CD86^+^DCs, CD8^+^T cells, and iNOS^+^M1-like TAMs. Meanwhile, the amount of immunosuppressive cells in the AptEM@CBA + NIR group was obviously reduced, such as MDSC and CD206^+^ M2-like TAMs. Such immune modulation efficacy was stronger than other controlling treatments which was absence of NIR, Apt or AMPCP, and the tendency kept consistence with that in the primary tumors. These results suggested that an efficient systemic anti-tumor immune response was generated to inhibit the distal tumor.

### Biosafety of AptEM@CBA for antitumor therapy

**Fig. 7:**
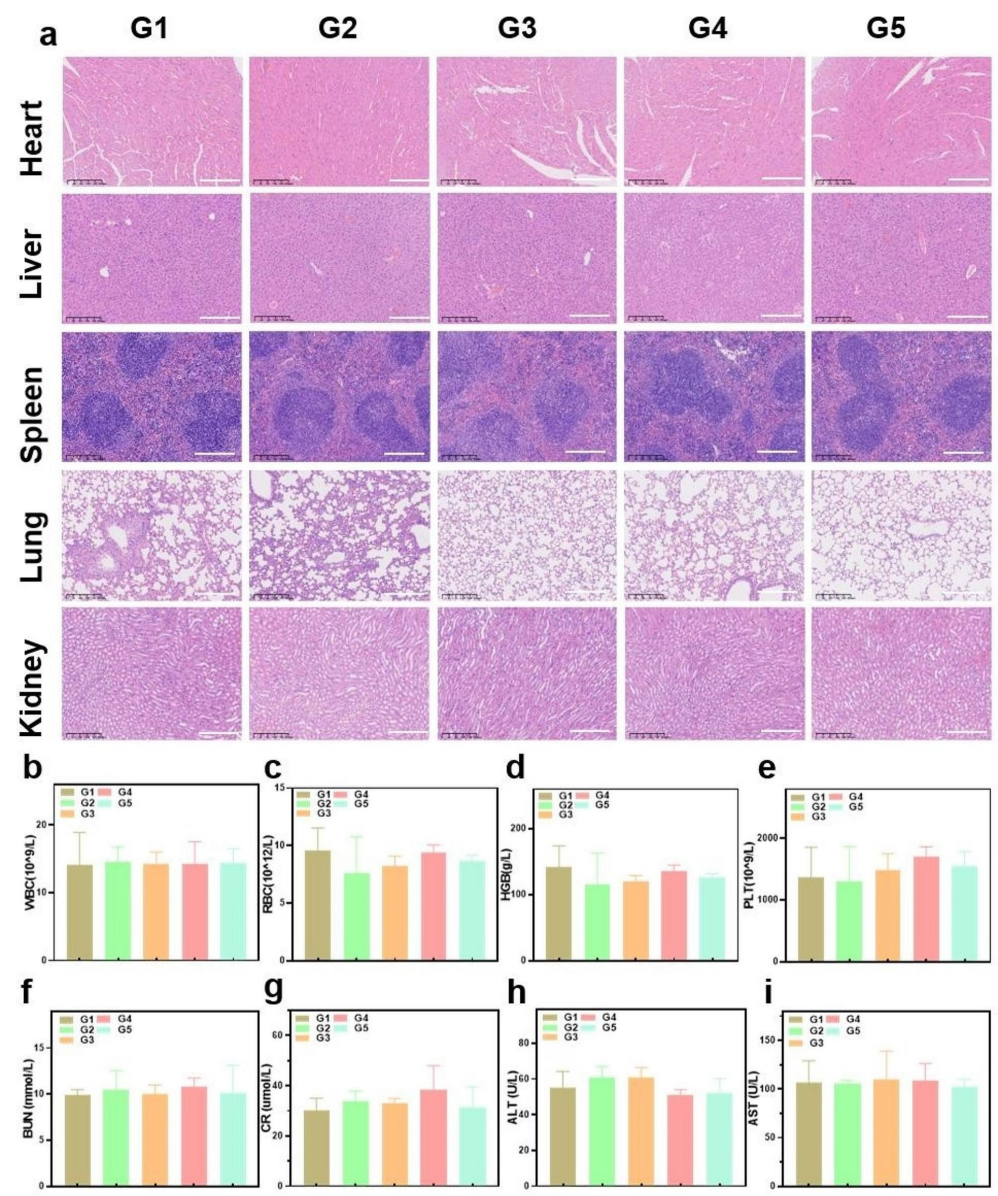
Biosafety evaluation of AptEM@CBA. (**a**) H&E staining of heart, liver, spleen, lungs, and kidneys stripped from each group of mice at the end of treatments. (**b**) WBC, (**c**) RBC, (**d**) HGB, and (**e**) PLT analysis of each group of mice at the end of the treatments. Serum levels of (**f**) BUN, and (**g**) CR, (**h**) ALT, and (**i**) AST for renal and hepatic function analysis. Scale bar: 200 μm. *n* = 4

The nanodrug’s biosafety was assessed in further detail. The tissues of the heart, liver, spleen, lung, and kidney were gathered to investigate whether AptEM@CBA plus NIR irradiation could cause damage to the primary organs. As the H&E staining displayed in Fig. 7a, the major organs of mice in each group maintained normal morphologies and did not suffer any obvious damages. Hematology examination revealed no statistically significant variations in several of critical blood indices, such as platelet (PLT), hemoglobin (HGB), red blood cell (RBC), and white blood cell (WBC), indicating that no infection or inflammation was brought on throughout the course of treatment. (Fig. 7b-e). Additionally, blood urea nitrogen (BUN), creatinine (CR), alanine aminotransferase (ALT), and aspartate aminotransferase (AST) levels in the serum further showed that there was no apparent damage to the liver or kidney (Fig. 7f-i). Collectively, the treatment of AptEM@CBA plus NIR irradiation achieved good biosafety in mice.

## Conclusion

In conclusion, we developed a new biomimetic CD73 blocking photothermal nanodrugs AptEM@CBA, composed by black phosphorus quantum dots, CD73 inhibitor AMPCP, chitosan nanogels, erythrocyte membrane and AS1411 aptamer, for effective photoimmunotherapy of lung cancer in mice. Attributed to the camouflage of the erythrocyte membrane and the modification of AS1411, good biocompatibility and tumor targeting ability were endowed to prolong the circulation time and tumoral enrichment of the nanodrug. Upon NIR irradiation, AptEM@CBA could fully utilize the photothermal ability of BPQDs and the CD73 inhibition function of AMPCP to realize efficient antitumor therapy and reduce negative outcomes. Animal-level therapeutic results showed that photothermal treatment combined with CD73 inhibition could effectively suppress LLC solid tumors and significantly revitalize systemic anti-tumor immune responses to inhibit distal tumor growth. Our work demonstrated the availability of photothermia combined with inhibition of adenosine generation for NSCLC treatment, extending the therapeutic options for lung cancer.

### Electronic supplementary material

Below is the link to the electronic supplementary material.

Supplementary Material 1

## Data Availability

No datasets were generated or analysed during the current study.

## Abbreviations

PTT: Photothermal therapy
ICD: Immunogenic cells death
BPQDs: Black phosphorus quantum dots
EM: Erythrocyte membrane
ICB: Immune checkpoint blockade
PD-1: Programmed cell death protein 1
PD-L1: Programmed cell death-ligand 1
TME: Tumor microenvironment
CRT: Calcium reticulin
DAMPs: Damage-associated molecular patterns
ATP: Adenosine triphosphate
CS: Chitosan
HMGB1: High mobility group protein B1
EE: Encapsulation efficiency

## References

[1] Liu W-J, Wang L, Zhou F-M, Liu S-W, Wang W, Zhao E-J, Yao Q-J, Li W, Zhao Y-Q, Shi Z (2023). Elevated NOX4 promotes tumorigenesis and acquired EGFR-TKIs resistance via enhancing IL-8/PD-L1 signaling in NSCLC. Drug Resist Update.

[2] Ozcan G, Singh M, Vredenburgh JJ (2023). Leptomeningeal Metastasis from Non-small Cell Lung Cancer and Current Landscape of treatments. Clin Cancer Res.

[3] Lahiri A, Maji A, Potdar PD, Singh N, Parikh P, Bisht B, Mukherjee A, Paul MK (2023). Lung cancer immunotherapy: progress, pitfalls, and promises. Mol Cancer.

[4] Zhu L, Liu J, Zhou G, Liu T-M, Dai Y, Nie G, Zhao Q (2021). Remodeling of Tumor Microenvironment by Tumor-Targeting Nanozymes enhances Immune activation of CAR T cells for combination therapy. Small.

[5] Li Z, Zhu L, Liu W, Zheng Y, Li X, Ye J, Li B, Chen H, Gao Y (2020). Near-infrared/pH dual-responsive nanocomplexes for targeted imaging and chemo/gene/photothermal tri-therapies of non-small cell lung cancer. Acta Biomater.

[6] Liu Y, Huang Y, Lu P, Ma Y, Xiong L, Zhang X, Yin Z, Xu H, Nie Y, Luo J (2023). Manganese Dioxide/Gold-based active Tumor Targeting Nanoprobes for enhancing photodynamic and low-temperature-photothermal combination therapy in Lung Cancer. ACS Appl Bio Mater.

[7] Chen ZH, Wei XQ, Zheng YR, Zhang ZW, Gu W, Liao WJ, Zhang H, Wang XY, Liu J, Li H, Xu W (2023). Targeted co-delivery of curcumin and erlotinib by MoS2 nanosheets for the combination of synergetic chemotherapy and photothermal therapy of lung cancer. J Nanobiotechnol.

[8] Liu T, Zhu M, Chang X, Tang X, Yuan P, Tian R, Zhu Z, Zhang Y, Chen X (2023). Tumor-specific photothermal-therapy-assisted Immunomodulation via Multiresponsive Adjuvant nanoparticles. Adv Mater.

[9] Sun K, Yu J, Hu J, Chen J, Song J, Chen Z, Cai Z, Lu Z, Zhang L, Wang Z (2022). Salicylic acid-based hypoxia-responsive chemodynamic nanomedicines boost antitumor immunotherapy by modulating immunosuppressive tumor microenvironment. Acta Biomater.

[10] Thompson EA, Powell JD (2021). Inhibition of the Adenosine Pathway to Potentiate Cancer Immunotherapy: potential for combinatorial approaches. Annu Rev Med.

[11] Zhu W, Dong Y, Xu P, Pan Q, Jia K, Jin P, Zhou M, Xu Y, Guo R, Cheng B (2022). A composite hydrogel containing resveratrol-laden nanoparticles and platelet-derived extracellular vesicles promotes wound healing in diabetic mice. Acta Biomater.

[12] Liu Y, Liu Y, Xu D, Zang J, Zheng X, Zhao Y, Li Y, He R, Ruan S, Dong H (2022). Targeting the negative feedback of Adenosine-A2AR metabolic pathway by a tailored Nanoinhibitor for Photothermal Immunotherapy. Adv Sci.

[13] Zhang C, Wang K, Wang H (2023). Adenosine in cancer immunotherapy: taking off on a new plane. Biochim Biophys Acta Rev Cancer.

[14] Giatromanolaki A, Kouroupi M, Pouliliou S, Mitrakas A, Hasan F, Pappa A, Koukourakis MI (2020). Ectonucleotidase CD73 and CD39 expression in non-small cell lung cancer relates to hypoxia and immunosuppressive pathways. Life Sci.

[15] Xu C, Jiang Y, Huang J, Huang J, Pu K (2021). Second Near-Infrared Light-Activatable Polymeric Nanoantagonist for Photothermal Immunometabolic Cancer Therapy. Adv Mater.

[16] Xiong H, Ma X, Wang X, Su W, Wu L, Zhang T, Xu Z, Sun Z-J (2021). Inspired epigenetic modulation synergy with Adenosine Inhibition elicits pyroptosis and Potentiates Cancer Immunotherapy. Adv Funct Mater.

[17] Guo T, Wu Y, Lin Y, Xu X, Lian H, Huang G, Liu J-Z, Wu X, Yang H-H (2018). Black Phosphorus Quantum dots with renal clearance property for efficient photodynamic therapy. Small.

[18] Kong N, Ji X, Wang J, Sun X, Chen G, Fan T, Liang W, Zhang H, Xie A, Farokhzad OC, Tao W (2020). ROS-Mediated selective killing effect of Black Phosphorus: mechanistic understanding and its Guidance for Safe Biomedical Applications. Nano Lett.

[19] Sun Z, Xie H, Tang S, Yu X-F, Guo Z, Shao J, Zhang H, Huang H, Wang H, Chu PK (2015). Ultrasmall Black Phosphorus Quantum dots: synthesis and use as Photothermal agents. Angew Chem-Int Edit.

[20] Pandey A, Nikam AN, Fernandes G, Kulkarni S, Padya BS, Prassl R, Das S, Joseph A, Deshmukh PK, Patil PO, Mutalik S (2021). Black phosphorus as Multifaceted Advanced Material nanoplatforms for potential Biomedical Applications. Nanomaterials.

[21] Jiang H, He Y, Zhao J, Chang R, He H, Li T, Zhang X, Shu B, Zhang W, Wang H (2023). Immunostimulant nanomodulator boosts antitumor immune response in triple negative breast cancer by synergism of vessel normalization and photothermal therapy. Nano Res.

[22] Chang R, Li T, Fu Y, Chen Z, He Y, Sun X, Deng Y, Zhong Y, Xie Z, Yang Y (2023). A PD-L1 targeting nanotheranostic for effective photoacoustic imaging guided photothermal-immunotherapy of tumor. J Mat Chem B.

[23] Zhao P, Xu Y, Ji W, Zhou S, Li L, Qiu L, Qian Z, Wang X, Zhang H (2021). Biomimetic black phosphorus quantum dots-based photothermal therapy combined with anti-PD-L1 treatment inhibits recurrence and metastasis in triple-negative breast cancer. J Nanobiotechnol.

[24] Xie Z, Peng M, Lu R, Meng X, Liang W, Li Z, Qiu M, Zhang B, Nie G, Xie N (2020). Black phosphorus-based photothermal therapy with aCD47-mediated immune checkpoint blockade for enhanced cancer immunotherapy. Light-Sci Appl.

[25] Huang D, Wu T, Lan S, Liu C, Guo Z, Zhang W (2022). In situ photothermal nano-vaccine based on tumor cell membrane-coated black phosphorus-Au for photo-immunotherapy of metastatic breast tumors. Biomaterials.

[26] Zhao Y, Xie Z, Deng Y, Huang A, He Y, Wen B, Liao X, Chang R, Zhang G, Zhu L (2022). Photothermal nanobomb blocking metabolic adenosine-A2AR potentiates infiltration and activity of T cells for robust antitumor immunotherapy. Chem Eng J.

[27] Li L, Zhang M, Liu T, Li J, Sun S, Chen J, Liu Z, Zhang Z, Zhang L (2022). Quercetin-Ferrum nanoparticles enhance photothermal therapy by modulating the tumor immunosuppressive microenvironment. Acta Biomater.

[28] Qiao L, Yang H, Gao S, Li L, Fu X, Wei Q (2022). Research progress on self-assembled nanodrug delivery systems. J Mat Chem B.

[29] Gu W, Meng F, Haag R, Zhong Z (2021). Actively targeted nanomedicines for precision cancer therapy: Concept, construction, challenges and clinical translation. J Control Release.

[30] Nam J, Son S, Park KS, Zou W, Shea LD, Moon JJ (2019). Cancer nanomedicine for combination cancer immunotherapy. Nat Rev Mater.

[31] Li P, Ruan L, Jiang G, Sun Y, Wang R, Gao X, Yunusov KE, Aharodnikau UE, Solomevich SO (2022). Design of 3D polycaprolactone/ ε-polylysine-modified chitosan fibrous scaffolds with incorporation of bioactive factors for accelerating wound healing. Acta Biomater.

[32] Liang Y, Wang Y, Wang L, Liang Z, Li D, Xu X, Chen Y, Yang X, Zhang H, Niu H (2021). Self-crosslinkable chitosan-hyaluronic acid dialdehyde nanoparticles for CD44-targeted siRNA delivery to treat bladder cancer. Bioact Mater.

[33] Caprifico AE, Foot PJS, Polycarpou E, Calabrese G (2020). Overcoming the blood-brain barrier: Functionalised Chitosan Nanocarriers. Pharmaceutics.

[34] Aranaz I, Alcantara AR, Concepcion Civera M, Arias C, Elorza B, Heras Caballero A, Acosta N (2021). Chitosan: an overview of its Properties and Applications. Polymers.

[35] Liu T, Xie Q, Dong Z, Peng Q (2022). Nanoparticles-based delivery system and its potentials in treating central nervous system disorders. Nanotechnology.

[36] Rao L, Zhao S-K, Wen C, Tian R, Lin L, Cai B, Sun Y, Kang F, Yang Z, He L (2020). Activating macrophage-mediated Cancer Immunotherapy by genetically edited nanoparticles. Adv Mater.

[37] Sheng S, Yu X, Xing G, Jin L, Zhang Y, Zhu D, Dong X, Mei L, Lv F (2023). An apoptotic body-based vehicle with Navigation for Photothermal-Immunotherapy by Precise Delivery and Tumor Microenvironment Regulation. Adv Funct Mater.

[38] Jin F, Qi J, Liu D, You Y, Shu G, Du Y, Wang J, Xu X, Ying X, Ji J, Du Y (2021). Cancer-cell-biomimetic Upconversion nanoparticles combining chemo-photodynamic therapy and CD73 blockade for metastatic triple-negative breast cancer. J Control Release.

[39] Krishnan N, Fang RH, Zhang L (2023). Cell membrane-coated nanoparticles for the treatment of cancer. Clin Transl Med.

[40] Shi G, Mukthavaram R, Kesari S, Simberg D (2014). Distearoyl Anchor-Painted erythrocytes with prolonged ligand Retention and circulation properties in vivo. Adv Healthc Mater.

[41] Fang RH, Hu C-MJ, Chen KNH, Luk BT, Carpenter CW, Gao W, Li S, Zhang D-E, Lu W, Zhang L (2013). Lipid-insertion enables targeting functionalization of erythrocyte membrane-cloaked nanoparticles. Nanoscale.

[42] Li J-Q, Zhao R-X, Yang F-M, Qi X-T, Ye P-K, Xie M (2022). An erythrocyte membrane-camouflaged biomimetic nanoplatform for enhanced chemo-photothermal therapy of breast cancer. J Mat Chem B.

[43] Xue X, Liu H, Wang S, Hu Y, Huang B, Li M, Gao J, Wang X, Su J (2022). Neutrophil-erythrocyte hybrid membrane-coated hollow copper sulfide nanoparticles for targeted and photothermal/anti-inflammatory therapy of osteoarthritis. Compos Pt B-Eng.

[44] Chen Z, Zeng Z, Wan Q, Liu X, Qi J, Zu Y (2022). Targeted immunotherapy of triple-negative breast cancer by aptamer-engineered NK cells. Biomaterials.

[45] Jain S, Deore SV, Ghadi R, Chaudhari D, Kuche K, Katiyar SS (2021). Tumor microenvironment responsive VEGF-antibody functionalized pH sensitive liposomes of docetaxel for augmented breast cancer therapy. Mater Sci Eng C-Mater Biol Appl.

[46] Xiao X, Ma Z, Li Z, Deng Y, Zhang Y, Xiang R, Zhu L, He Y, Li H, Jiang Y (2023). Anti-BCMA surface engineered biomimetic photothermal nanomissile enhances multiple myeloma cell apoptosis and overcomes the disturbance of NF-KB signaling in vivo. Biomaterials.

[47] Lv H, Wang T, Ma F, Zhang K, Gao T, Pei R, Zhang Y (2022). Aptamer-functionalized targeted siRNA delivery system for tumor immunotherapy. Biomed Mater.

[48] Yang Z, Gao D, Guo X, Jin L, Zheng J, Wang Y, Chen S, Zheng X, Zeng L, Guo M (2020). Fighting Immune Cold and Reprogramming Immunosuppressive Tumor Microenvironment with Red Blood Cell membrane-camouflaged nanobullets. ACS Nano.

[49] Shao X, Ding Z, Zhou W, Li Y, Li Z, Cui H, Lin X, Cao G, Cheng B, Sun H (2021). Intrinsic bioactivity of black phosphorus nanomaterials on mitotic centrosome destabilization through suppression of PLK1 kinase. Nat Nanotechnol.

[50] Khademi Z, Lavaee P, Ramezani M, Alibolandi M, Abnous K, Taghdisi SM (2020). Co-delivery of doxorubicin and aptamer against Forkhead box M1 using chitosan-gold nanoparticles coated with nucleolin aptamer for synergistic treatment of cancer cells. Carbohydr Polym.

[51] Jain A, Thakur K, Sharma G, Kush P, Jain UK (2016). Fabrication, characterization and cytotoxicity studies of ionically cross-linked docetaxel loaded chitosan nanoparticles. Carbohydr Polym.

[52] Chen Q, Jia C, Xu Y, Jiang Z, Hu T, Li C, Cheng X (2022). Dual-pH responsive chitosan nanoparticles for improving in vivo drugs delivery and chemoresistance in breast cancer. Carbohydr Polym.

