# Supplementary material for: Biomimetic nanodrug blocks CD73 to inhibit adenosine and boosts antitumor immune response synergically with photothermal stimulation
Source: J Nanobiotechnology. 2024 Apr 30;22:214. doi: 10.1186/s12951-024-02487-4 (PMC11059694; doi:10.1186/s12951-024-02487-4)
Supplement: Supplementary file 1 — Supplementary Material 1 [file 12951_2024_2487_MOESM1_ESM.docx]

Biomimetic Nanodrug Blocks CD73 to Inhibit Adenosine and Boosts Antitumor Immune Response Synergically with Photothermal Stimulation

Tan Li^†^, Xingyu Zhang^†^, Chengyu Shi, Qiao Liu, Yuetao Zhao*

T. Li, C. Shi, X. Zhang, Q. Liu, Prof. Y. Zhao

Department of Biochemistry and Molecular Biology, School of Life Sciences, Central South University, Changsha, 410012, China.

^†^Tan Li and Xingyu Zhang contributed equally to this work.

^*^Correspondence:

Yuetao Zhao E-mail: ytzhao@csu.edu.cn


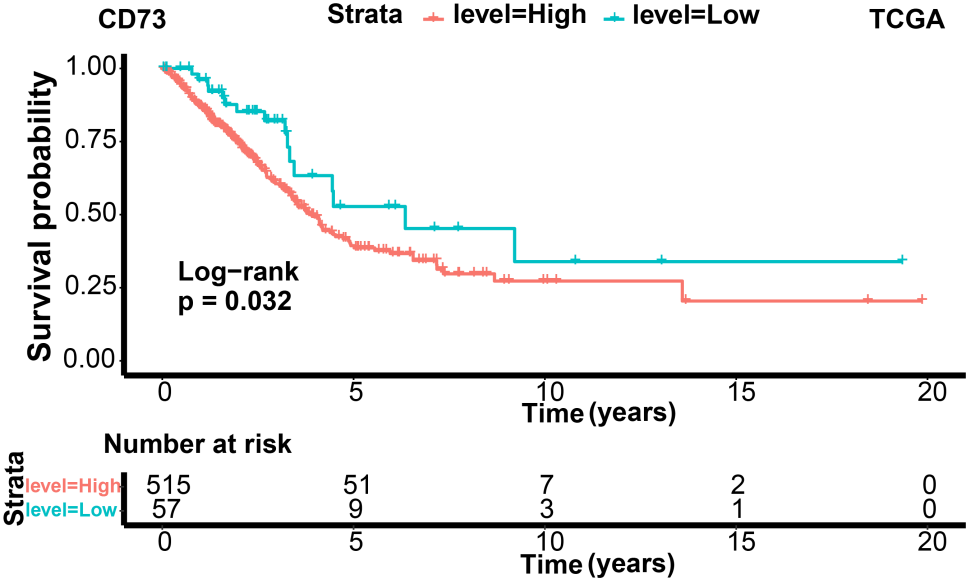


**Figure S1.** To understand the relationship between CD73 expression and the prognosis of NSCLC patients, we downloaded NSCLC-related RNA-seq data and clinical survival data from the TCGA database, and analyzed the relationship between CD73 expression and the prognosis of NSCLC patients by using the "survival" package in R language (p < 0.05 was considered significant).


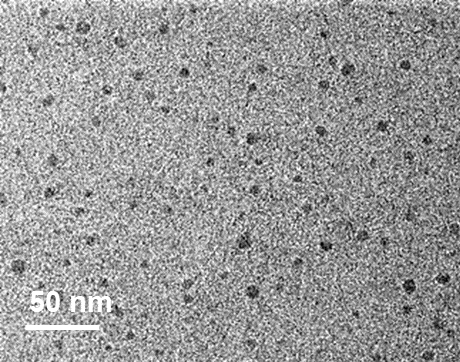


**Figure S2.** TEM of black phosphorus quantum dots (BPQDs).

**Figure S3.** AMPCP encapsulation efficiency（EE) in AptEM@BA (without chitosan nanogel) and AptEM@CBA (with chitosan nanogel). n = 3. ***p*<0.01


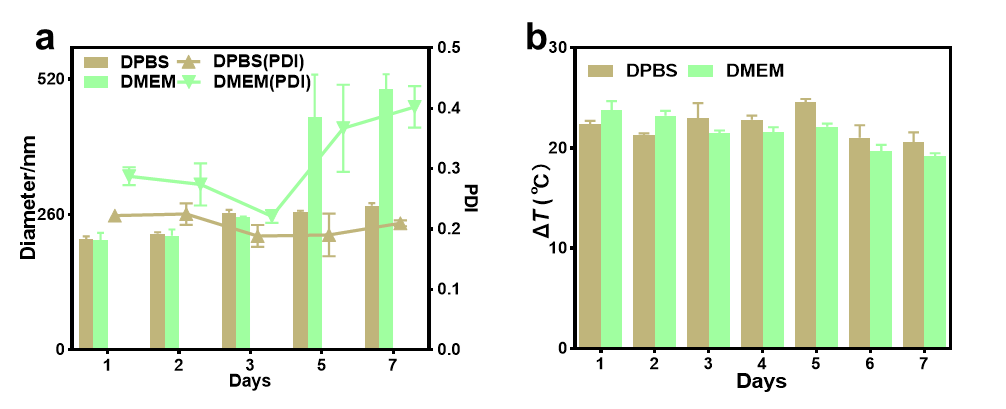


**Figure S4.** Stability of AptEM@CBA in different solutions. a) Particle size, and b) Photothermal properties of AptEM@CBA in DPBS and DMEM over seven days. n = 3


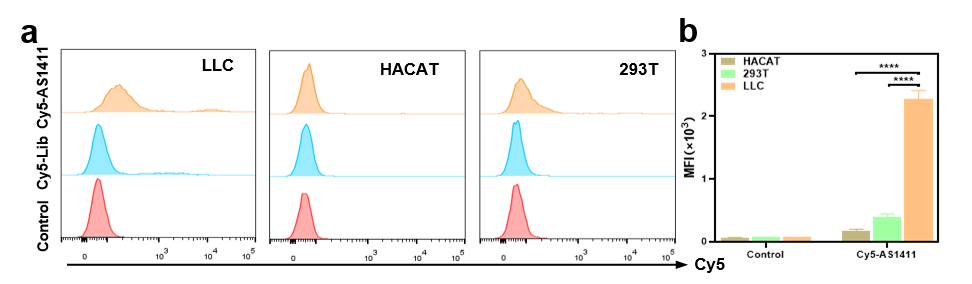


**Figure S5.** Cellular targeting of Cy5-AS1411. a) Flow cytometric, b) quantitative analysis of Cy5-1411 targeting to LLC, HACAT, and 293T cells. n = 3. *****p*<0.0001

**Figure S6.** Quantitative analysis of targeting ability of AS1411 to different cells. n = 3. ***p*<0.01

**Figure S7.** Quantitative analysis of cellular phagocytosis for AptEM@CBA in different cells. n = 3. ***p*<0.01; ****p*<0.001


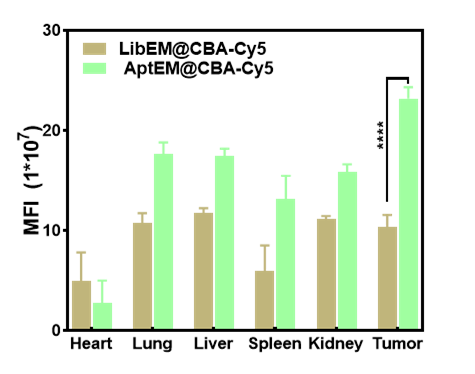


**Figure S8.** Quantitative analysis of AptEM@CBA enrichment in tumor sites 12 hours after injection. n = 3. *****p*<0.0001


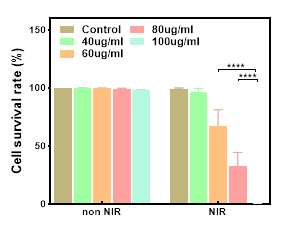


**Figure S9.** Quantitative analysis of in vitro photothermal ablation of tumor cells by AptEM@CBA. n = 3. *****p*<0.0001


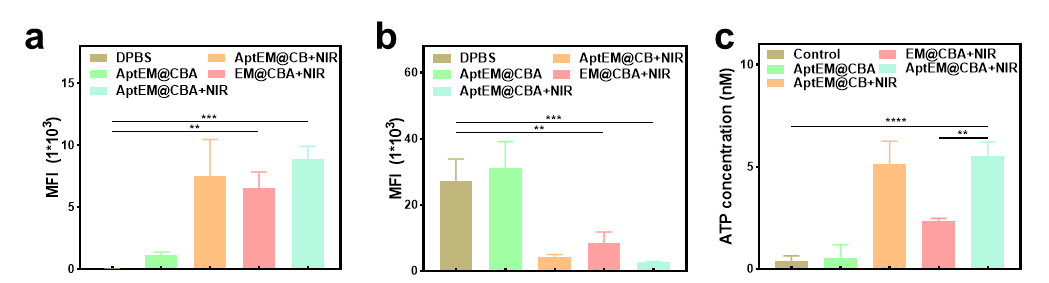


**Figure S10.** Quantitative analysis of a) CRT immunofluorescence; b) HMGB1 immunofluorescence, and c) ATP release. n=3. ***p*<0.01; ****p*<0.001; *****p*<0.0001

**Figure S11**. Quantitative analysis of flow cytometric analysis of MHC-Ⅱ for DC maturation induction *in vitro*. n=3. ****p*<0.001


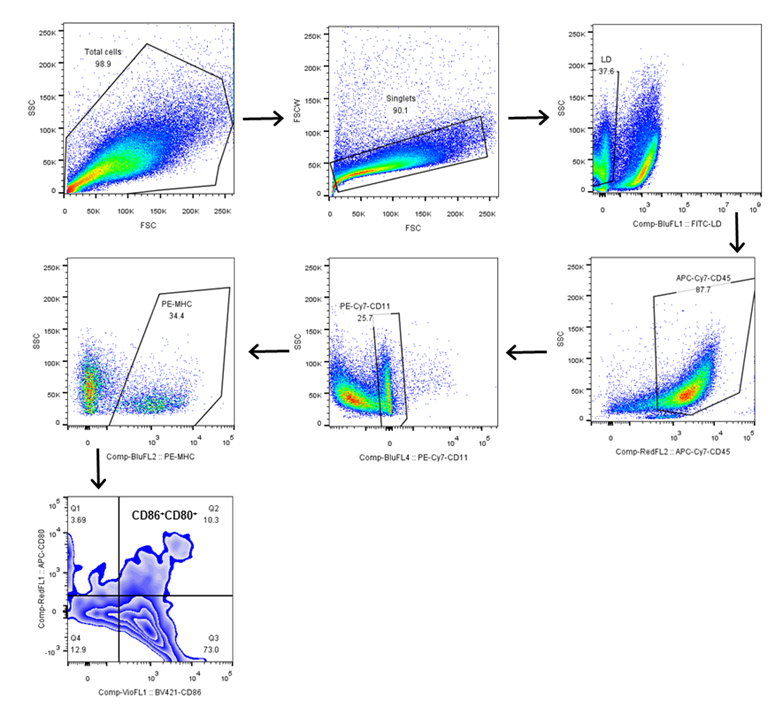


**Figure S12**: The gating strategy for the flow cytometric analysis of in vitro DC maturation in Figure 4f.


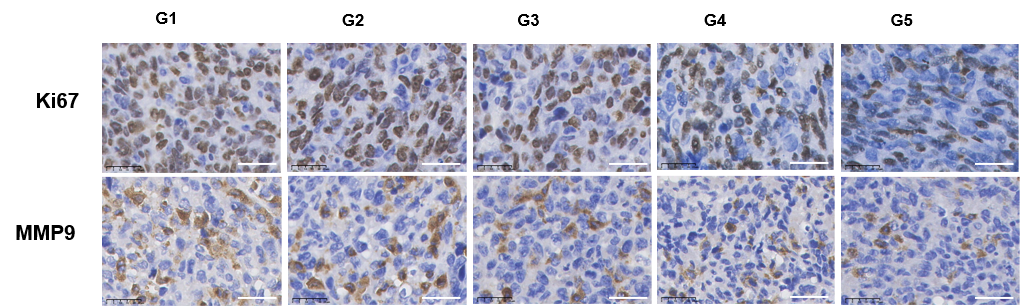


**Figure S13.** Representative immunohistochemical staining images of Ki67 and MMP9 for each group (scar bar, 25 um). G1: DPBS, G2: AptEM@CBA, G3: AptEM@CB + NIR, G4: EM@CBA + NIR, G5: AptEM@CBA + NIR.


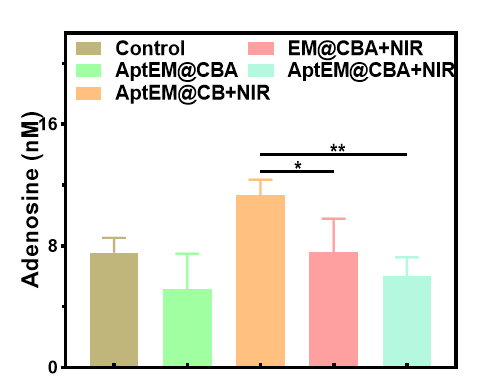


**Figure S14.** Tumor adenosine levels in the primary tumor tissues. n = 4. **p*<0.05; ***p*<0.01


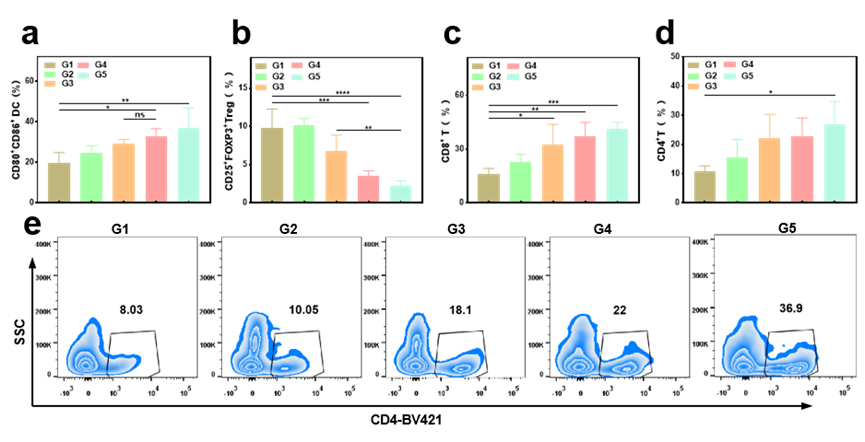


**Figure S15.** Statistical analysis of a) CD80^+^CD86^+^ DC, b) CD25^+^FOXP3^+^ Tregs, c) CD8^+^ T cells and d) CD4^+^T cells for each group by GraphPad Prism. e) Representative plots of flow cytometric analysis for CD4^+^T cells. G1: DPBS, G2: AptEM@CBA, G3: AptEM@CB + NIR, G4: EM@CBA + NIR, G5: AptEM@CBA + NIR. n = 3. **p*<0.05; ***p*<0.01; ****p*<0.001; *****p*<0.0001


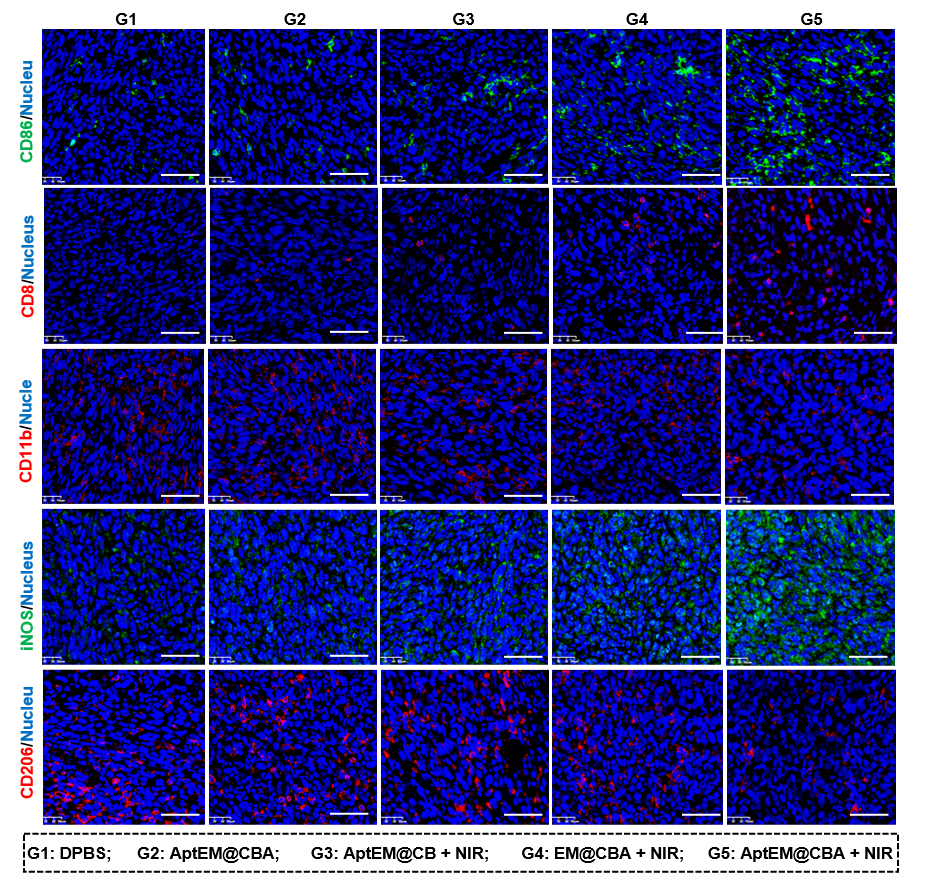


**Figure S16.** Representative immunofluorescence images of CD86^+^ DCs, CD8^+^ T cells, CD11b^+^ MDSCs, iNOS^+^ M1-type TAMs and CD206^+^ M2-type TAMs in distal tumor tissues. Scale bar: 50 μm.

**
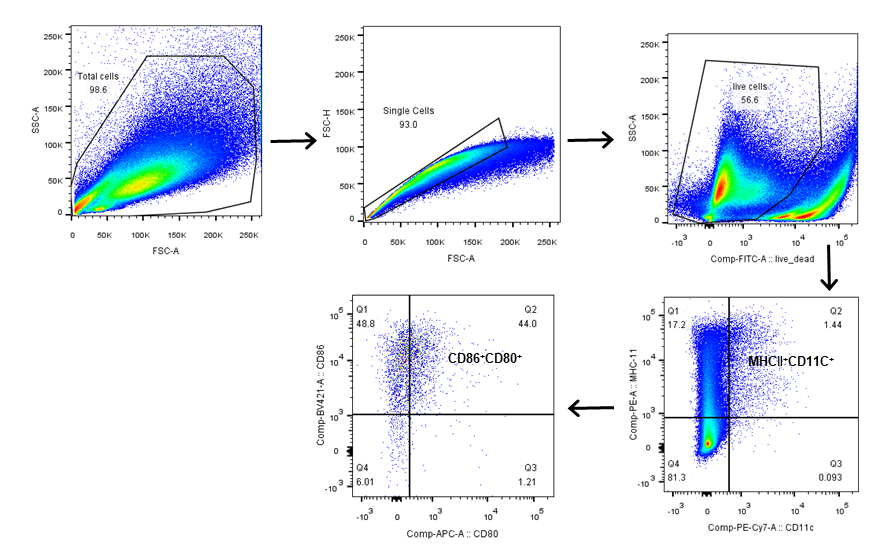
** **Figure S17:** The gating strategy for the flow cytometric analysis of CD80^+^CD86^+^ DCs in Figure 6d.

**
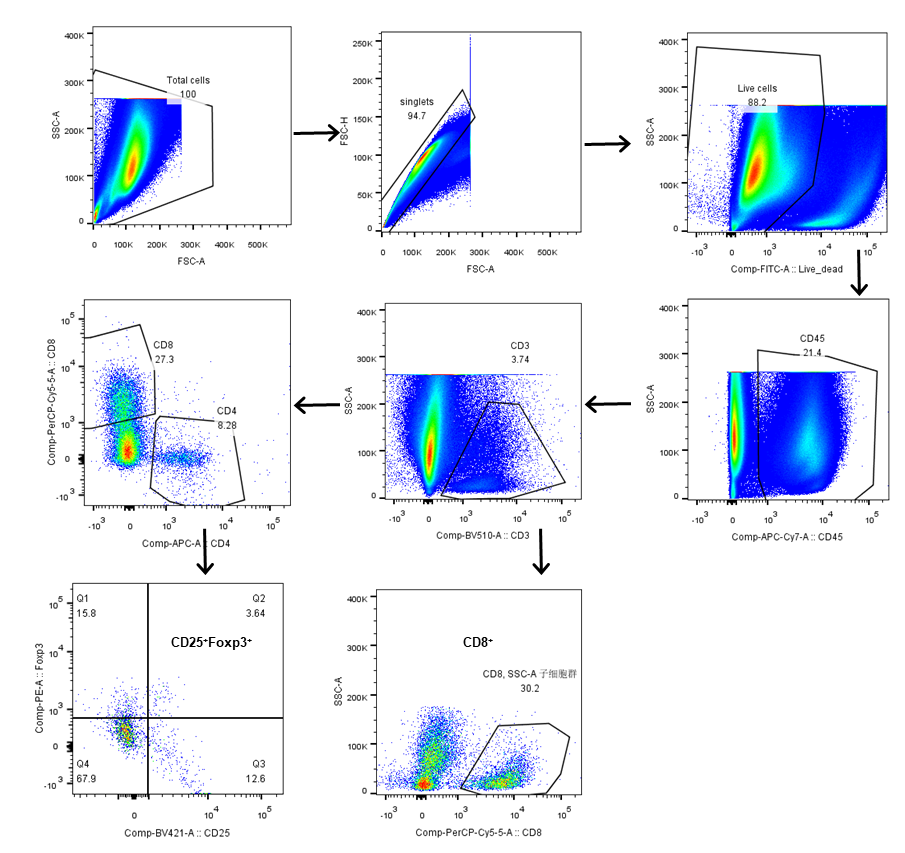
**

**Figure S18:** The gating strategy for the flow cytometric analysis of CD25^+^FOXP3^+^ Tregs and CD8^+^ T cells in Figure 6e-f.
